# Predicting the microalgae lipid profile obtained by supercritical fluid extraction using a machine learning model

**DOI:** 10.3389/fchem.2024.1480887

**Published:** 2024-10-25

**Authors:** Juan David Rangel Pinto, Jose L. Guerrero, Lorena Rivera, María Paula Parada-Pinilla, Mónica P. Cala, Gina López, Andrés Fernando González Barrios

**Affiliations:** ^1^ Grupo de Diseño de Productos Y Procesos (GDPP), Department of Chemical and Food Engineering, Universidad de los Andes, Bogotá, Colombia; ^2^ Metabolomics Core Facility—MetCore, Vice-Presidency for Research, Universidad de los Andes, Bogotá, Colombia; ^3^ Unidad de Saneamiento y Biotecnología Ambiental (USBA), Departamento de Biología, Facultad de Ciencias, Pontificia Universidad Javeriana (PUJ), Bogotá, Colombia

**Keywords:** supercritical fluid extraction, regression models, lipidomic, COSMO-SAC, extremophile microalgae

## Abstract

In this study a Machine Learning model was employed to predict the lipid profile from supercritical fluid extraction (SFE) of microalgae *Galdieria* sp. USBA-GBX-832 under different temperature (40, 50, 60°C), pressure (150, 250 bar), and ethanol flow (0.6, 0.9 mL min^-1^) conditions. Six machine learning regression models were trained using 33 independent variables: 29 from RD-Kit molecular descriptors, three from the extraction conditions, and the infinite dilution activity coefficient (IDAC). The lipidomic characterization analysis identified 139 features, annotating 89 lipids used as the entries of the model, primarily glycerophospholipids and glycerolipids. It was proposed a methodology for selecting the representative lipids from the lipidomic analysis using an unsupervised learning method, these results were compared with Tanimoto scores and IDAC calculations using COSMO-SAC-HB2 model. The models based on decision trees, particularly XGBoost, outperformed others (RMSE: 0.035, 0.095, 0.065 and coefficient of determination (R^2^): 0.971, 0.933, 0.946 for train, test and experimental validation, respectively), accurately predicting lipid profiles for unseen conditions. Machine Learning methods provide a cost-effective way to optimize SFE conditions and are applicable to other biological samples.

## 1 Introduction

### 1.1 Lipids extraction techniques

Lipids are a diverse group of biomolecules, generally classified into eight categories (fatty acyls, glycerolipids, glycerophospholipids, sphingolipids, sterol lipids, prenol lipids, saccharolipids and polyketides), based on their hydrophobic or amphipathic properties and chemically functional backbones (Fahy et al., 2005; Liebisch et al., 2020). Traditionally, oleaginous plants and seeds have been the primary sources of lipids for biofuels production. In recent years, microalgae have gained attention for their potential to provide a diverse range of bioactive molecules. In particular, extremophilic microalgae have the ability to grow under extreme conditions such as acidic or alkaline pH, high temperatures, light and heavy metal concentrations. Some microalgae lipids, such as polyketides and prenol lipids, are reported to possess antioxidant, anti-inflammatory, cytotoxic, and even anticancer properties (De Luca et al., 2021; Khan et al., 2018; Castro et al., 2023). Furthermore, glycerophospholipids, known for their amphiphilicity, are effective emulsifying agents, stabilizing oil-water emulsions in delivery systems for cosmetic and pharmaceutical industries (Li et al., 2019). This shift towards microalgae is due to their rapid growth rates, high lipid content, and adaptability to various environments (De Luca et al., 2021; Khan et al., 2018; Castro et al., 2023).

Obtaining lipids involves different standard methodologies that include mechanical cell disruption and solvent extraction. Currently there are different techniques that use solvents, one of the most used is Bligh and Dyer (B&D) method for lipid quantitation at analytical level (Bligh and Dyer, 1959; Azmin et al., 2016). However, the reliance of B&D method on methanol and chloroform presents environmental and health risks unsuitable for industrial applications (Santoro et al., 2019). Other organic solvents like ethanol, dichloromethane, dimethyl ether, and hexane have been studied but often yield lower results compared to the B&D method, and some of these solvents may be toxic and hazardous pollutants, unsuitable for cosmetic, pharmaceutical and food industries (de Jesus et al., 2019; Cauchie et al., 2021; Xiao et al., 2012).

Soxhlet extraction offers improved extraction yields, however, large volumes of solvents required can be expensive to remove, and thermal degradation may also occur caused by the extraction performed at the boiling point of the solvent for extended periods of time (Akyil et al., 2018). Alternative methods such as microwave-assisted extraction, ultrasound-assisted extraction, and supercritical fluid extraction (SFE) are efficient, fast and sustainable. However, their application has been limited due to the higher capital investment for complex equipment (Bligh and Dyer, 1959; Chang et al., 2017; Desgrouas et al., 2014; Zeković et al., 2017; Orio et al., 2012).

### 1.2 Extraction of lipids employing supercritical fluid extraction (SFE)

Supercritical fluid extraction (SFE) is green technology that is growing for obtaining bioactive compounds because it is capable of solubilizing lipophilic substances in shorter process time, and the solvent can be easily removed from the final extract: this ensures minimal alteration of the bioactive metabolites and preserves their biological functional properties. It achieves high selectivity by tuning pressure and temperature conditions. Its main disadvantage is the high cost of equipment compared to other extraction techniques (Crampon et al., 2013).

Over 90% of SFE processes use supercritical carbon dioxide (scCO2) due to its low critical temperature (31°C) and pressure (74 bar), non-flammability, non-toxicity and low cost (Capuzzo et al., 2013; Reid et al., 1988). Besides, CO2 is a gas in atmospheric conditions, achieving almost complete CO2 removal in extracts and resulting in solvent-free extract (Molino et al., 2020). scCO2 exhibits high diffusivity and low viscosity, similar to gasses, which allows the solvent phase to penetrate into the biological matrix, while its high density, like liquids, provides good solvating power. Together these properties enhance the penetration in the biological matrix and the solubilization of the intracellular compounds. However, CO2’s non-polarity limits its solvent effectiveness, showing affinity only to non-polar compounds (de Melo et al., 2014). Cosolvents such as ethanol or isopropanol are used to modify the solvent polarity (Yousefi et al., 2019).

Extraction temperature and pressure significantly affect the compounds solubility in the solvent phase, depending on the chemical properties of the target compounds. In SFE, efficiency increases with both pressure and temperature. However, higher temperature and pressure can increase solubility of all compounds, even unwanted by-products, such as waxes or chlorophylls. This reduces extraction specificity and necessitates additional purification steps. Morcelli et al. reported reduced target compound yields due to increased chlorophyll concentrations when extracting carotenoids from *Chlorella sorokiniana* at higher pressure and temperature (Morcelli et al., 2021).

Additionally, higher temperatures may cause thermal degradation of compounds, while higher pressure can increase fluid density and obstruct diffusivity into the biomass, decreasing extraction yields (Molino et al., 2020; de Melo et al., 2014; Yousefi et al., 2019). This thermal degradation and reduced yield at higher pressures were reported by Sanzo et al. when extracting astaxanthin and lutein from *Haematococcus pluvialis* (Sanzo et al., 2018)*.* Thus, many researchers aim to find optimal extraction conditions to maximize the yield and bioactivity of extracts (Sanzo et al., 2018; Macías-Sánchez et al., 2010; Nobre et al., 2006; Macias Sanchez et al., 2009; Machmudah et al., 2006; Santoyo et al., 2006).

### 1.3 Thermodynamics-based methods for modeling supercritical fluid extraction

Developing an experimental design to identify optimal extraction conditions considering all variables involves significant time and resource investment. Researchers have formulated accurate and reliable models considering thermodynamics and kinetic constraints, equilibrium relationships, and mass transfer mechanisms across a spectrum of temperatures, pressures, and phase compositions (Izadifar and Abdolahi, 2006). These models are classified into three categories: empirical equations, analogical models drawing parallels between heat and mass transfer, and models derived from integrated differential mass balances (Sodeifian et al., 2016).

Empirical equation-based models fit for specific and limited cases, while heat and mass transfer models aim to describe extraction process robustly but are constrained by highly idealized assumptions, such as isothermal processes or homogeneous mixtures. These assumptions often overlook factors like particle size effects or cell wall rupture dynamics (Rai et al., 2014).

Thermodynamics-based models, such as those using the activity coefficient, describe non-ideal mixtures (Atkins, 2006). The activity coefficient indicates solvent-solute affinity and extraction efficiency. Models like UNIFAC use group-contribution methods to estimate interaction parameters by breaking molecules into functional groups, facilitating broader generalization and reducing experimental workload (Fredenslund et al., 1977). However, these models have some inherent disadvantages: require extensive experimental data for accurate fragmentation, struggle with nonadditive molecular effects, and offer limited insight into solute-solvent interactions, which hinders their practical utility (Klamt, 1995).

**FIGURE 1:**
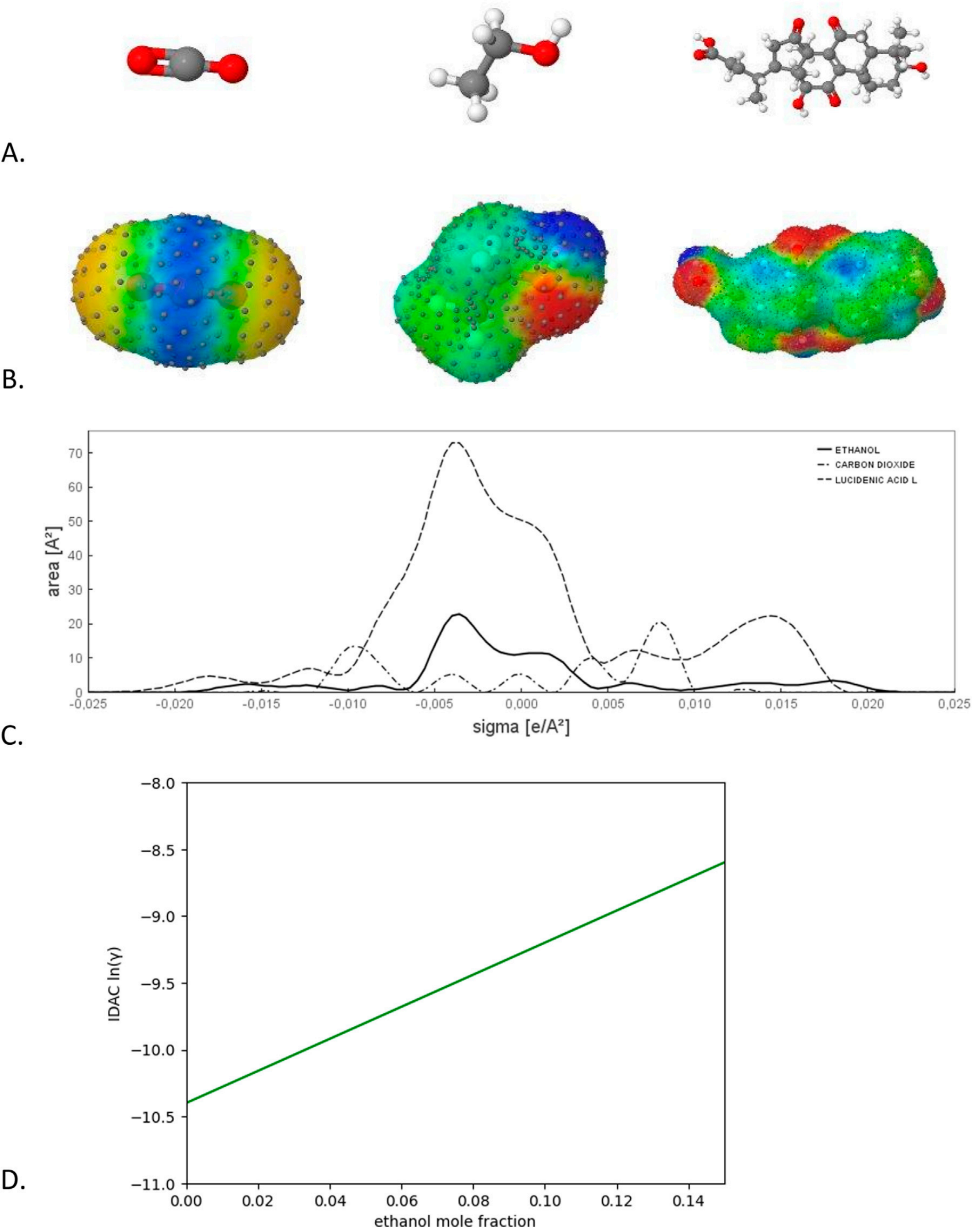
Step calculation with a COSMO-based model. **(A)** 3D molecular structure of the molecules in the mixture: carbon dioxide, ethanol and lipid lucidenic acid L. **(B)** molecular surface charge distribution calculation. In red a high electronic density, in blue a low electronic density. This is the most time-consuming step due to quantum mechanical calculations. **(C)** Bidimensional projection of the surface charge distributions into sigma-profiles. **(D)** Determining the thermodynamic property, infinite dilution activity coefficient (IDAC) of the lipid calculated at 50°C, varying ethanol mole fraction. Figures A, B, and C were obtained using JCOSMO-2.9.12.

An alternative to group-contribution models is the conductor-like screening model (COSMO), which relies on computational quantum mechanics. Unlike UNIFAC-Modified (2002), which uses 612 fitting parameters related to size, shape, and functional group interactions, COSMO models require only four universal parameters. These models predict thermo-physical properties without experimental data and calculate the chemical potential of any molecule in any mixture (Gerber and Soares, 2010; Lin and Sandler, 2001). Figure 1 presents the step-by-step calculation with a COSMO-based model, starting with the 3D molecular structures, and finishing with the calculation of thermodynamic properties under temperatures and compositions in the extraction system. COSMO-based models have been used successfully for predicting the optimal temperature and ethanol composition for SFE to obtain carotenoids. However, those calculations are based on individual lipids against CO2-ethanol mixtures and cannot account for solute competition or positive synergies that may enhance the extraction yields (Morcelli et al., 2021). To address these limitations, a comprehensive model is needed, incorporating not only COSMO calculations but also other chemoinformatics tools to accurately describe these effects.

### 1.4 Molecular descriptors

For decades, researchers have sought to translate the encoded information in chemical structures into numerical representations that computers can understand and manipulate (Wang et al., 2021). This effort led to the development of Quantitative Structure-Activity Relationship (QSAR) approaches, a powerful *in silico* method. QSAR establishes quantitative relationships between a molecule’s structure (represented by molecular descriptors) and its properties, including biological activities, reaction mechanisms, and physicochemical properties, such as solubility (Willighagen, 2010).

Over 5,000 molecular descriptors have been proposed, capturing various aspects of a molecule’s structure (Consonni and Todeschini, 2010). These descriptors range from basic features like the number and types of atoms to more detailed information such as connectivity, geometry, charge distribution, and hydrogen bonding potential (Grisoni et al., 2018).

### 1.5 Machine learning in SFE

The proliferation of Artificial Intelligence (AI) in recent years has been remarkable, permeating various sectors and becoming an integral part of daily activities (Prezhdo, 2020). AI applications are now used as personal assistants, customer preference predictors, and creators of images and natural language (Mistry et al., 2021). The success of machine learning in the technology sector is anticipated to be similar in science. The exponential increase in computational power over the past 2 decades has enabled *in silico* investigations previously deemed unfeasible due to limited time and experimental resources.

Physics-driven tools have emerged, facilitating high-throughput computational screening for drug discovery, predicting molecular properties based on Quantitative Structure-Property Relationships (QSPRs), and calculating activity coefficients for thermodynamic systems using quantum mechanics models (Winter et al., 2023). In contrast, machine learning operates without relying on an understanding of underlying physics, leveraging vast datasets to make predictions. This paradigm shifts from physics-driven to data-driven modeling has seen various machine learning algorithms implemented across diverse scientific disciplines, including chemistry, biology, fluid dynamics, and material science (Butler et al., 2018).

Research in supercritical fluids has also embraced machine learning, from molecular simulation to estimation of solubilities in supercritical conditions (Roach et al., 2023). In the domain of SFE, there is significant interest in optimizing processes. Much of the analysis has focused on predicting extraction yield under various conditions, employing complex algorithms such as artificial neural networks (ANN), adaptive neuro fuzzy inference system (ANFIS) or cascade-forward back-propagation network (CFBPN) to address an optimization problem (Ghoreishi and Heidari, 2013; Heidari and Ghoreishi, 2013; Lashkarbolooki et al., 2013; Ghoreishi et al., 2016; Idris et al., 2022; Valim et al., 2018). Studies have also investigated the solubility of different organic compounds in scCO2, but these have often focused on individual molecules or a limited set of compounds (Kamali and Mousavi, 2008; Nguyen et al., 2022; Huwaimel and Alobaida, 2022; Kostyrin et al., 2022; Aminian and ZareNezhad, 2020). There is a noticeable absence of studies aiming to generalize the solubility of hundreds of organic compounds in a solvent or to elucidate changes in lipid profile composition based on SFE variables (Roach et al., 2023).

Consequently, in the present study, six Machine Learning models were tested to predict the microalgae lipid profile obtained by SFE at different pressure, temperature and ethanol flow conditions. The lipid profile of the extracts was elucidated using RP-LC-ESI(+/−)-QTOF-MS platform, and K-Medoids, an unsupervised learning method, was used for systematic lipid selection.

## 2 Materials and methods

### 2.1 Dataset compilation

**FIGURE 2:**
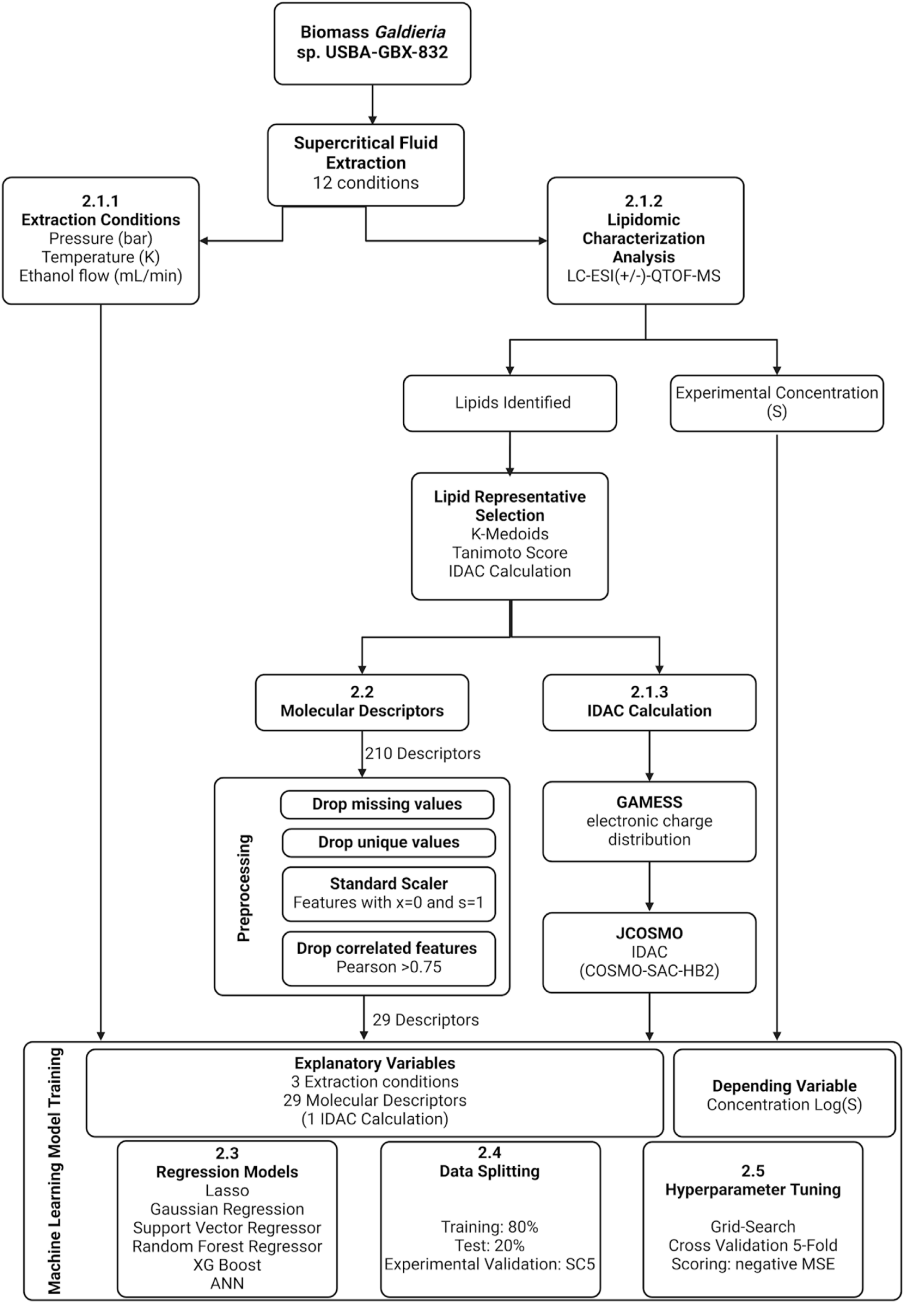
Data Flow Diagram of Lipid Extraction and Lipidomic Characterization from *Galdieria* sp. and Machine Learning Regression Models Training for Recovery Prediction.

The data flow for building the models is presented in Figure 2. A single dataset consolidated all the information for training and testing the models. The defined extraction conditions, the cleaned molecular descriptors and the results from IDAC calculations served as independent variables, while the lipid recovery, measured in the lipidomic characterization analysis, was the dependent variable. Some experiments were performed to collect all this information, and some intermediate steps were necessary for preprocessing the collected data. The data, files and codes used along the methodology have been made available in a GitHub repository (https://github.com/Grupo-de-Diseno-de-Productos-y-Procesos/Lipids-SFE).

#### 2.1.1 Conditions for supercritical fluid extraction (SFE) of microalgae *Galdieria* sp. USBA-gbx-832

The algal strain *Galdieria* sp. USBA-GBX-832 in lyophilized pellets was obtained from culture cultivation at Pontificia Universidad Javeriana, Colombia (CMPUJ U832). This biomass was cultured in mixotrophic conditions in MG911 during 8 days maintaining at 43±2°C, consistent agitation speed of 170 rpm, light intensity of 20 umol. m⁻^2^s⁻^1^, and aeration rate of 0.2 vvm (López et al., 2019; Rivera, 2024). The biomass of *Galdieria* sp. USBA-GBX-832 was frozen for 24 h at −80°C and freeze-dried (Alpha 1-2 LDPlus, Martin Christ, Germany) at a pressure of 4 × 10^−4^ and temperature of −40°C for 48 h. To ensure uniformity, biomass underwent homogenization before the extraction process. SFE experiments employed carbon dioxide (99.99% purity, Messer, Colombia) and ethanol (99.8%, ITW Reagents, Germany) as solvents.

SFE extractions were performed using the MV-10 ASFE System (Waters, United States) following the manufacturer’s recommendations. Freeze-dried biomass was powdered with mortar with pestle and sieved, selecting particle size between 180 and 500 μm, and dried at 45°C for 12 h to eliminate moisture. Samples of 1.0 g of microalgae biomass were wrapped with filter paper (7–10 µm pore size) and placed in the extraction vessels. Extraction conditions (pressure, temperature, CO2 flow, cosolvent flow, and extraction time) were controlled *via* the panel, with a CO2 flow rate of 5 mL/min for 75 min. Pressure (150 and 250 bar ±1 bar), temperature (40, 50, and 60°C ± 0.5°C), and cosolvent flow (0.6 and 0.9 mL/min of ethanol ±0.1 mL/min) were varied, based on literature reports (de Melo et al., 2014). Extracts were collected in amber flasks to prevent daylight degradation, concentrated in a vacuum concentrator (Vacufuge^®^ Plus, Eppendorf) at 40°C for 3 h, and freeze-dried (Alpha one to two LDPlus, Martin Christ) at −20°C, 1 mbar for 26 h. Lipidomic analysis was conducted on 10 µg samples of each extraction (see Table 1 for experimental design).

**TABLE 1:** Experimental conditions defined for supercritical fluid extraction.

Temperature ethanol flow pressure (bar)	40°C		50°C		60°C	
	0.6 mLmin^-1^	0.9 mLmin^-1^	0.6 mLmin^-1^	0.9 mLmin^-1^	0.6 mLmin^-1^	0.9 mLmin^-1^
150	SC1	SC2	SC3	SC4	SC5	SC6
250	SC7	SC8	SC9	SC10	SC11	SC12

#### 2.1.2 Lipidomic characterization analysis and representative lipid selection

Lipidomic characterization was conducted using RP-LC-ESI (+/−)-QTOF-MS. Supercritical extracts were dissolved in MeOH:MTBE (1:1) until obtaining a solution at 200 ppm. Samples were vortexed and centrifuged at 13,000 rpm for 10 min to 4°C. Chromatographic elution was achieved by injecting 2 µL of sample into InfinityLab Poroshell C18 column (3.0 × 100 mm 2.7 µm) at flow rate of 0.6 mLmin^-1^, with a column temperature of 60°C. Mobile phases consisted of 10 mM ammonium formate, ACN:H2O (60:40) and 0.1% of formic acid for phase A and 10 mM in ammonium formate, IPA:ACN (90:10) and 0.1% of formic acid for phase B and gradient elution:0–2 min, 15%–30% B; 2–2.5 min 30%–48% B; 2.5–11 min, 48%–82% B; 11–11.5 min, 82%–99% B; 11.5–12 min, 99% B; 12–12.1 min, 99%–15% By 12.1–18 min, 15% B. The mass spectrometer was operated in positive mode (ESI +/−) with a range of 65–1700 m/z. Capillary voltage was set to 3,000, the drying gas flow rate was 12 L min^-1^ at 250°C, gas nebulizer 3.59 bar (52 psi), fragmentor voltage 175 V, skimmer 65 V and octopole radio frequency voltage (OCT RF vpp) 750 V. Data were collected in centroid mode at a scan rate of 1.02 spectra per second. For electrospray ionization in positive mode, two reference masses were used: m/z 121.0509 [C5H4N4+H]+ and m/z 922.0098 [C18H18O6N3P3F24 + H]+. For electrospray ionization negative mode were used: m/z 112.9856 [C2O2F3 (NH4)], m/z 1,033.9881 (C18H18O6N3P3F24).

The Lipidomic characterization process is limited in identifying all lipids at the highest level of detail and several lipids share the same shorthand notation. Full structural information is required for further calculations, needing a detailed description of the identified lipids. To address this, a methodology was developed for selecting a representative lipid from the available reported lipids. First, candidate names and structural information in isomeric SMILES format were obtained from Lipid MAPS (Lipid Maps, 2024). Next, molecular descriptors were calculated using the RDKit 2023.9.4 library, from the 210 descriptors available in RDKit, 29 were selected following the methodology explained in section 2.2. The K-Medoids clustering algorithm, an unsupervised learning method, grouped the candidate lipids, and for each group, a centroid was calculated using the cleaned molecular descriptors data. The lipid closest to this centroid was selected as the representative lipid.

The results of this methodology were compared and analyzed against those obtained through Tanimoto similarity scores and IDAC calculations. Tanimoto scores were computed for each pair of candidates, and the mean score for each candidate relative to the others in the group was calculated. The highest-scoring candidate (closest to 1.0) was considered the most structurally similar lipid within the group. The IDAC calculation methodology (further details in the next section) involved evaluating each candidate’s activity coefficients under the SFE conditions. The candidate exhibiting the lowest squared error against the mean results of the group was identified as possessing the representative physical and thermodynamic behavior under SFE conditions.

#### 2.1.3 Infinite dilution activity coefficient (IDAC) evaluation

The calculation of IDAC requires information about the electronic charge distribution of the molecules involved in the CO2-ethanol-lipid thermodynamic system (see Figure 1). The electronic charge distribution of lipids was determined using GAMESS software (Mark Gordon’s Quantum Theory Group, de Iowa State University, United States) (Barca et al., 2020), with support from COSPRT patch routine developed by The Virtual Laboratory for Properties Prediction (LVPP, UFRGS, Brazil) (Soares et al., 2020). For these calculations, 3D-structural information in MOL file format is necessary. The resulting files, in GOUT format, were integrated into the compounds’ library of JCOSMO 2.9.12 (LVPP, UFRGS, Brazil) (Ferrarini et al., 2018). This software was used to calculate IDAC, at the same SFE conditions, temperature and ethanol mole fraction. The lipids were set at a mole fraction of 1 × 10^−5^ to ensure infinite dilution conditions.

### 2.2 RDKit molecular descriptors selection

A set of 210 molecular descriptors was calculated using the RDKit 2023.9.4 library. Data preprocessing involved a Python 3.10 script that removed descriptors with significant missing or unique values. Pearson correlation analysis was then performed with a threshold of 0.75 to reduce redundancy. A final set of 29 descriptors was selected for training the regression models. The descriptors selected by this methodology are specified in Supplementary Data 1.

### 2.3 Machine learning models description

Six Machine Learning regression models were trained and tested: Lasso (Tibshirani, 1996), Gaussian Regression (GR) (Rasmussen and Williams, 2006), Support Vector Machines (SVR) (Smola and Schölkopf, 2004), Random Forest (RFR), Gradient-Boosted Trees (XGBoost) (Freund and Schapire, 1997), and Artificial Neural Network (ANN) (Atienza, 2018). These algorithms were implemented using Python 3.10.12, Keras library was used for ANN, and Scikit-Learn library for the other methods.

### 2.4 Data splitting

The dataset with three extraction conditions, 29 molecular descriptors, IDAC results, and the recovery of the lipids, in logarithmic scale, were split randomly into training and testing sets with an 80:20 ratio. Data from the extraction conditions SC5 were set aside from the beginning and excluded from the training and testing data sets. This information was used to validate the models’ capacity to predict the lipid profile under new, unseen extraction conditions.

### 2.5 Hyperparameter tuning and evaluation of the models

The models were trained and evaluated both including and excluding the IDAC calculations to assess the influence of this variable in the performance of the regression models. A hyperparameter tuning was performed for every model using Grid Search with 5-fold Cross-Validation (See Supplementary Data 2). After training, the following metrics were calculated: Root Mean Squared Error (RMSE), Mean Absolute Error (MAE), and coefficient of determination (R^2^) (Scikit Learn, 2024). Hyperparameter tuning process, training and tests calculations were performed using the Python library Scikit-Learn (Pedregosa et al., 2012).

## 3 Results and discussion

### 3.1 Lipid profile of the supercritical extracts

**FIGURE 3:**
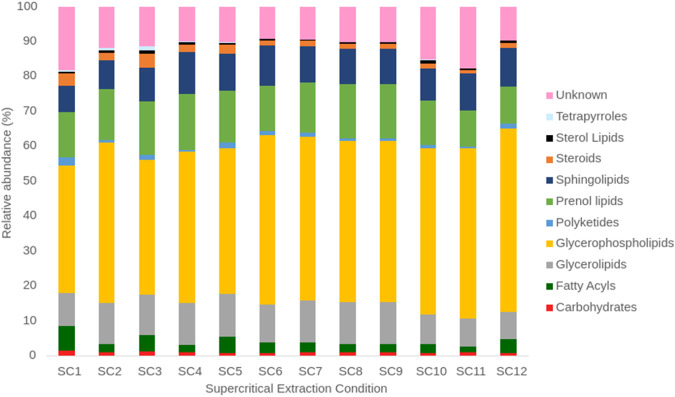
Relative abundance of different lipid classes in the twelve supercritical extracts.

A total of 139 features were identified from the supercritical extracts, 89 could be annotated while 50 remain unknown. Supplementary Data 3 provides a comprehensive list of all the identified lipids along with their recovery under the 12 extraction conditions. The lipidomic characterization revealed the primary components extracted from microalgae *Galdieria* sp. USBA-GBX-832 were lipids with glycerol backbone: glycerophospholipids and glycerolipids; followed by sphingolipids, prenols and fatty acyls (Figure 3). Although triglycerides had previously been identified in the microalgae under the cultivation conditions from which the biomass was obtained, none were detected in the supercritical extracts (Rivera, 2024).

Most of the lipids were identified in all the extracts; however, it is observed that, depending on the condition employed, their abundances were different. Figure 3 shows the differences in lipid class profile for each extract. For instance, at lower pressure (150 bar), more fatty acyls are extracted when the ethanol flow is lower (0.6 mLmin^-1^) compared to higher flow (0.9 mLmin^-1^). This suggests that fatty acyls are less attracted to the solvent when the polarity increases. Interestingly, this difference is less noticeable at higher pressure (250 bar), indicating that pressure helps dissolve fatty acyls, making the CO2-ethanol mixture a more effective solvent. In contrast, the abundance of glycerophospholipids increase as all three variables increase. The lowest abundance is observed at SC1, while the highest abundance is at SC12. At low pressure, the cosolvent has a stronger effect for glycerolipids than the observed with fatty acyls.

### 3.2 Representative lipid selection using an unsupervised method

Supplementary Data 4 shows all the lipids identified in the lipidomic characterization.

analysis, with their corresponding lipid annotated. In cases where more than one lipid was reported with the same shorthand notation, the selection was performed using the unsupervised algorithm K-Medoids as was explained above.

**FIGURE 4:**
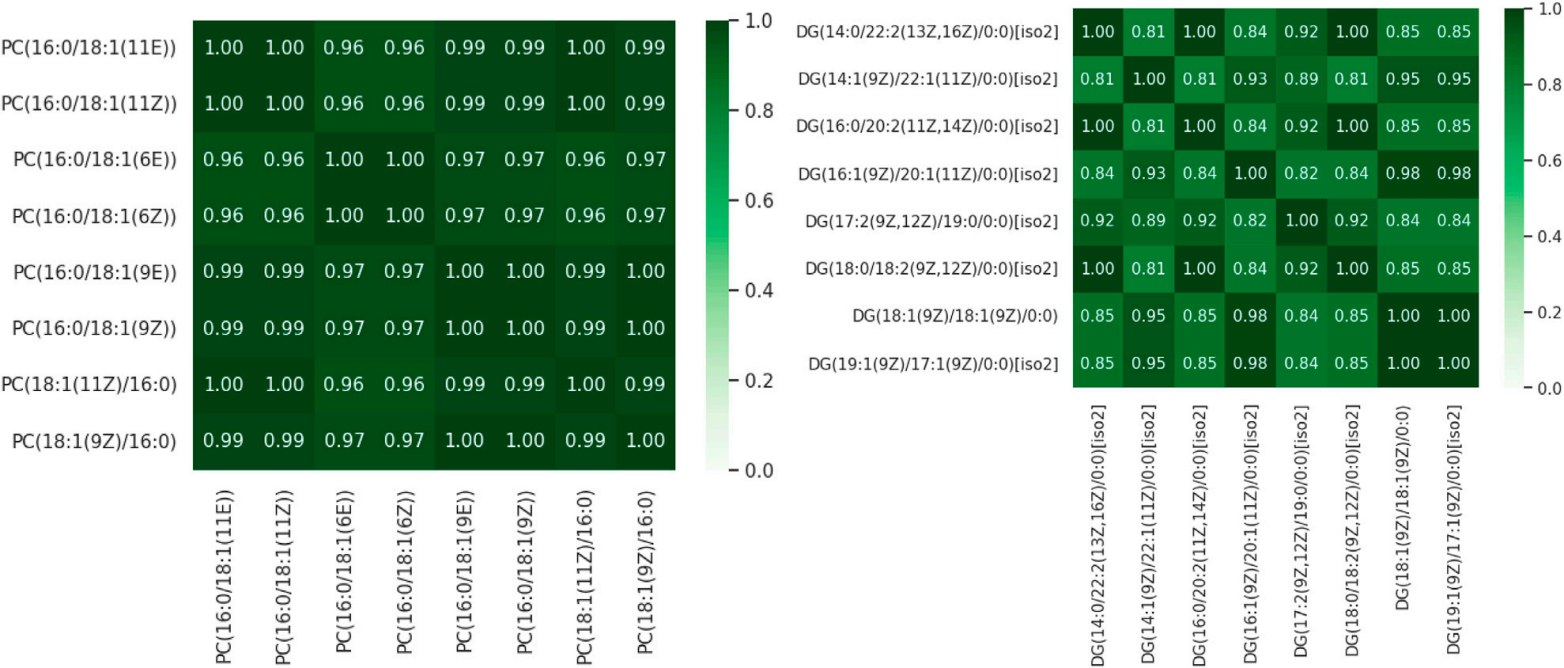
Tanimoto Score for pairs of PC 16:0_18:1 **(A)** and DG 36:2 **(B)** candidates.

The representative lipid selection results were compared with Tanimoto scores and IDAC calculations. In Figure 4 can be observed the results of Tanimoto score calculations. High similarity scores (>0.80, and in some cases >0.95) were observed when comparing the candidates. This high similarity can be attributed to the minimal structural differences between the candidates, primarily involving the location of double bonds. Furthermore, candidates were ranked based on their average score, revealing that no candidate stood out significantly as all had nearly identical values.

**FIGURE 5:**
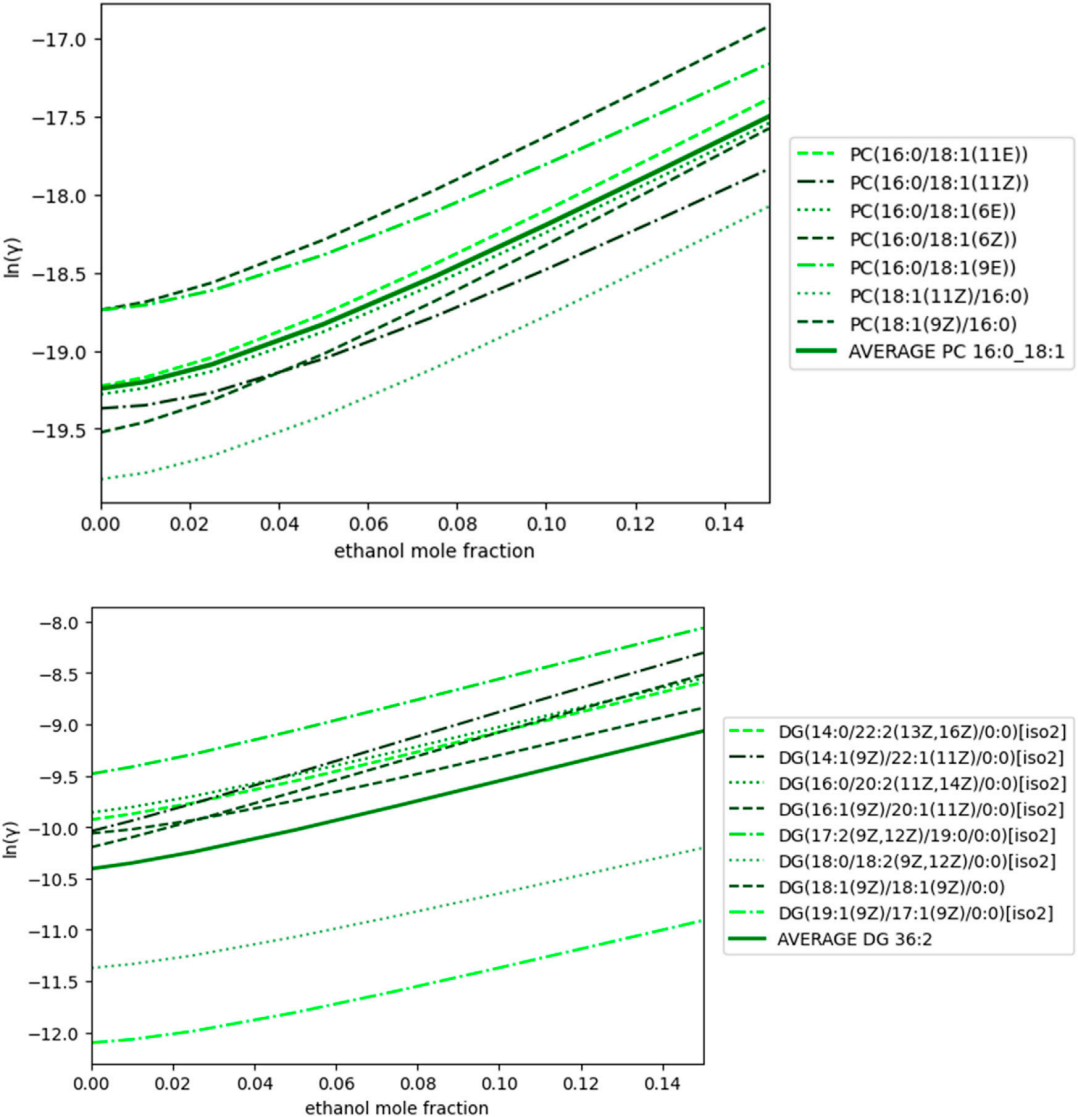
IDAC calculations for PC 16:0_18:1 **(A)**, DG 36:2 **(B)** candidates at 50°C as a function of the mole fraction of ethanol.

Additionally, all candidates for each lipid exhibit the same trend and order of magnitude when calculating IDAC (See Figure 5). These findings, combined with the Tanimoto Score results, suggest that while the representative lipid selection through the unsupervised learning method may introduce uncertainty, the physical and thermodynamic behavior of any candidate would correspond to the behavior observed experimentally in the context of extraction (Complementary results in Supplementary Data 5). K-Medoids and Tanimoto Score give a quick result, while IDAC calculation is highly time-consuming, calculating a single molecule’s surface-charge distribution can take several days of computer processing. It is important to mention that the only way to validate the selected lipid is experimentally, either through standard solutions or by enhancing the detection capacity of the instruments. However, both options are unfeasible for this work, and generally for most research endeavors.

### 3.3 Model performance and prediction over the experimental dataset

The lipidomic characterization produced a dataset of 1,056 entries. Additionally, 210 molecular descriptors were calculated. The cleaning data and dimension reduction process was performed by removing variables with missing or unique values, and high correlations. This step aimed to reduce the computational cost and noise, prevent overfitting and improve generalization. The final set of 29 molecular descriptors, combined with the extraction conditions (pressure, temperature, and ethanol flow rate), serve as input for training the selected Machine Learning algorithms for predicting lipid concentration under the given extraction conditions. Table 2 shows the regression metrics for all the assessed models.

**TABLE 2:** Performance metrics of the assessed Machine Learning algorithms.

Model	MSE train	MSE test	MSE validation	RMSE train	RMSE test	RMSE validation	R2 train	R2 test	R2 validation
Lasso-	0.805	0.953	0.808	0.648	0.908	0.653	0.463	0.355	0.453
Lasso+	0.624	0.657	0.597	0.389	0.432	0.356	0.702	0.679	0.723
GR-	0.243	0.388	0.329	0.059	0.150	0.108	0.951	0.894	0.910
GR+	0.231	0.426	0.325	0.054	0.181	0.105	0.959	0.865	0.918
XGB-	0.186	0.308	0.254	0.035	0.095	0.065	0.971	0.933	0.946
XGB+	0.179	0.367	0.288	0.032	0.135	0.083	0.992	0.917	0.914
RF-	0.134	0.310	0.291	0.018	0.096	0.084	0.985	0.933	0.927
RF+	0.137	0.394	0.324	0.019	0.155	0.105	0.986	0.884	0.918
SVR-	0.273	0.378	0.251	0.074	0.143	0.063	0.937	0.905	0.953
SVR+	0.233	0.400	0.290	0.054	0.160	0.084	0.958	0.881	0.934
ANN-	0.271	0.381	0.260	0.074	0.145	0.067	0.939	0.897	0.944
ANN+	0.210	0.534	0.309	0.044	0.285	0.095	0.966	0.788	0.926

Abbreviations: +, including IDAC, as variable; -, excluding IDAC, as variable; GR, gaussian regression; XGB, XG, boost; RF, random forest; SVR, support vector regressor; ANN, artificial neural network.

Testing the predictiveness of these models on unseen data revealed some limitations. The Lasso displayed the worst performance due to its reliance on linear regression. For instance, while Gaussian Regression exhibited excellent performance on the training set (R^2^ ≈ 0.998), it showed a notable drop when tested on unseen data (R^2^ < 0.85). This overfitting was reduced with manual hyperparameter tuning, raising the test performance to around R^2^ ≈ 0.90 (see Supplementary Data 2). Models based on decision tree architectures, such as Random Forest and XG Boost, consistently demonstrated better performance and generalization. While XGBoost showed promising results with low MSE and RMSE values on training, test, and experimental validation data, it is essential to note that some models, including ANN and SVR, struggled to achieve R^2^ > 0.90 and RMSE <0.1 on the test data.

Comparing the performances of the models with and without IDAC, notably, the Lasso model achieved consistently coefficient of determination around 0.7 for training, test and validation data. This suggests a strong correlation between solubility and activity coefficient, but it is still insufficient for training an accurate model based on linear regression. For the other models, overfitting is observed. While training performance improved when including IDAC, this did not translate to test and validation data. This drop was especially significant for Random Forest and ANN. These results indicate that although IDAC is related to solubility, it is not essential for building a robust model that predicts the lipid profile of the extracts.

Further analysis of the variables identified two molecular descriptors, Quantitative Estimation of Drug-likeness (qed) and Minimum Electrotopological State Index (MinEStateIndex) were highly correlated with IDAC (Pearson correlation coefficients of 0.76 and 0.78, respectively). This redundancy between features might be causing the overfitting. The qed measure reflects the underlying distribution of molecular properties including molecular weight, logP, topological polar surface area, meanwhile the MinEStateIndex calculates the minimum electro-topological state value across all atoms in a molecule. This value can help assess the overall electron-withdrawing character of the molecule. Both descriptors are related to the activity coefficient calculation.

One limitation in calculating IDAC using COSMO-SAC-HB2 is that it does not account for pressure as a variable. The newer COSMO-SAC-Phi model addresses this by incorporating pressure into the IDAC calculations [72]. However, to do so, saturation data is required to compute the parameters involved in the activity coefficient under varying pressures. Unfortunately, this saturation data is currently unavailable for the identified lipids, as these complex molecules lack sufficient experimental data in existing databases.

**FIGURE 6:**
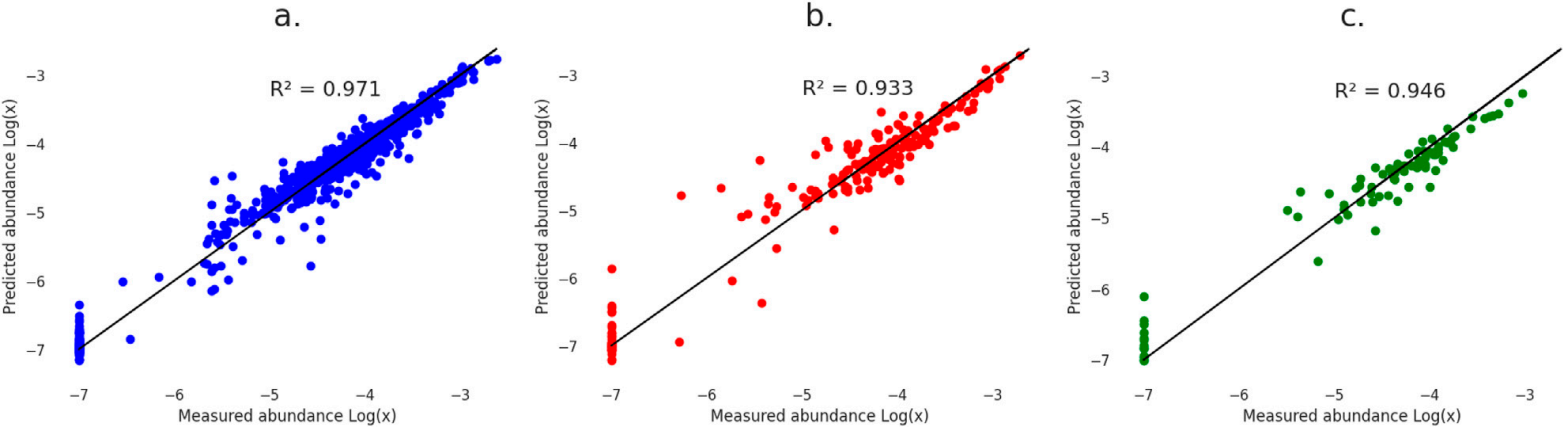
Regression results for XG Boost model using **(A)** Training data, **(B)** Test data, and **(C)** Validation data from SC5.

Figure 6 presents the regression results using the best-performing model, XGBoost, applied to the training, test, and experimental validation data. The low MSE and RMSE values, along with the high coefficient of determination score for the validation data indicate that the model can accurately predict a complete lipid profile for unseen extraction conditions. Moreover, the model-maintained accuracy when predicting for intermediate experimental conditions. Graphical results for the other models are available in Supplementary Data 6.

All models exhibited high uncertainty when predicting no lipid recovery under specific extraction conditions, particularly for lipids recovered only in SC1. To retain valuable information, lipids with no recovery (relative abundance of 0.00 in Supplementary Data 3) were assigned an arbitrary Log [x] value of −7 during the logarithmic transformation. This value, chosen to be lower than the smallest detected abundance, represented a lipid quantity too low for detection by the instruments. XGBoost outperformed the other models in handling these low-recovery lipids, although predicted values still remained very low (Log [x] < −6).

The relatively small dataset of 1,056 entries, coupled with the specific experimental conditions under which it was generated, may limit the model’s ability to generalize beyond its current scope. Despite this, the model demonstrated strong predictive performance by accurately forecasting the complete lipid profile concentration under a combination of conditions unseen by the model during training. This experimental validation suggests the model’s reliability within the dataset’s context, even though the validation data originated from the same experimental design that fed the training process.

Although the proposed methodology could be extended to different biological samples, including other microalgae species beyond *Galdieria* sp. USBA-GBX-832, the current model is specifically trained on data unique to this species. As a result, its ability to generalize to other microalgae remains uncertain, with predicted lipid profiles closely tied to the biological and cultivation characteristics of *Galdieria sp*. To enhance generalization, additional data reflecting their distinct biological properties and environmental conditions of other microalgae species would be required. Future research should prioritize testing the model on a broader range of species to assess its adaptability and refine it for improved cross-species prediction.

## 4 Conclusion

In this work, a Machine Learning approach was used to build the first model capable of accurately predicting the complete lipid profile during supercritical fluid extraction across a range of temperatures and cosolvent flow conditions. Additionally, a systematic approach for representative lipid selection was developed, demonstrating that, in an extraction context, the chosen lipids will exhibit physical and thermodynamic behavior observed experimentally.

The Lasso model with IDAC demonstrated the strong correlation between solubility and the activity coefficient, although the other models that include IDAC suffered overfitting. The best performing model for predicting the lipid profile of the extract was XG Boost without IDAC. IDAC results were limited to the thermodynamic model used, COSMO-SAC-HB2, which does not consider pressure effects. A COSMO-based model that does consider pressure, COSMO-SAC-Phi, was not used because the necessary saturation information was unavailable.

Although the build model is restricted for predicting the lipid profile of the microalgae, this methodology allows researchers to reduce the cost and time needed to identify the desired extraction conditions, whether to achieve the highest extraction yield or to optimize the recovery of specific lipids or lipid groups. For instance, the model can help pinpoint conditions that maximize the extraction of valuable lipids like phosphoglycerolipids or reduce the presence of undesired compounds like chlorophylls.

## Data Availability

The datasets presented in this study can be found in online repositories. The names of the repository/repositories and accession number(s) can be found below: https://github.com/Grupo-de-Diseno-de-Productos-y-Procesos/Lipids-SFE.
