# Supplementary material for: Predicting the microalgae lipid profile obtained by supercritical fluid extraction using a machine learning model
Source: Front Chem. 2024 Oct 25;12:1480887. doi: 10.3389/fchem.2024.1480887 (PMC11543471; doi:10.3389/fchem.2024.1480887)
Supplement: Supplementary file 1 [file DataSheet4.pdf]

Supplementary Data 4

Representative lipids selected using an unsupervised learning method

Compound	Molecular Formula	Molecular weight (DB) g/mol	Isomers	ID Level	Lipid selected
Carbohydrates and carbohydrate conjugates					
Armillarin	$C_{24}H_{30}O_6$	414.2042	1	MS/MS	Armillarin
Fatty Acyls					
aminopentanoic acid	$C_5H_{11}NO_2$	117.079	6	Putative	4-amino-pentanoic acid
FAL 16:0	$C_{16}H_{32}O$	240.2453	1	MS/MS	Palmitaldehyde
FA 22:5;O	$C_{22}H_{34}O_3$	346.2508	1	MS/MS	Leiopathic acid
FA 22:6;O	$C_{22}H_{32}O_3$	344.2351	1	MS/MS	13-HDoHE
FA 22:4;O	$C_{22}H_{36}O_3$	348.2664	1	MS/MS	12,15-epoxy-13,14-dimethyleicosa-12,14,16-trienoic acid
FA 26:1;O2	$C_{26}H_{50}O_4$	426.3709	1	MS/MS	Hexacosanedioic acid
FOH 5:1	$C_5H_{10}O$	86.0732	5	Putative	3-Methyl-2-buten-1-ol
Stearamide	$C_{18}H_{37}NO$	283.2875	1	MS/MS	Stearamide
NA 23:4	$C_{23}H_{39}NO$	345.3032	8	Putative	N-(2-methyl-2S-hydroxy-ethyl) arachidonoyl amine
NA 25:4	$C_{25}H_{43}NO$	373.3345	13	Putative	N-(2S-hydroxy-propyl) alpha,alpha-dimethylarachidonoyl amine
6E-Heneicosen-11-one	$C_{21}H_{40}O$	308.3079	1	MS/MS	6E-Heneicosen-11-one
12-Nonadecen-9-one	$C_{19}H_{36}O$	280.2766	1	MS/MS	12Z-Nonadecen-9-one
WE 24:2;O4	$C_{24}H_{44}O_6$	428.3138	1	MS/MS	Glyceryl lactooleate
Glycerolipids					
MGDG 16:0_18:1	$C_{43}H_{80}O_{10}$	756.5752	1	MS/MS	MGDG(16:0/18:1(9Z))
MGDG 16:0_18:2	$C_{43}H_{78}O_{10}$	754.5595	1	MS/MS	MGDG(16:0/18:2(9Z,12Z))
MGDG 18:2_18:2	$C_{45}H_{78}O_{10}$	778.5595	1	MS/MS	MGDG(18:2(9Z,12Z)/18:2(9Z,12Z))
MGDG 18:1_18:1	$C_{45}H_{82}O_{10}$	782.5908	1	MS/MS	MGDG(18:1(9Z)/18:1(9Z))
MGDG 36:6	$C_{45}H_{74}O_{10}$	774.5282	1	MS/MS	MGDG(18:3(9Z,12Z,15Z)/18:3(9Z,12Z,15Z))

continued on next page

Supplementary Data 4. (*Continued*)

Compound	Molecular Formula	Molecular weight (DB) g/mol	Isomers	ID Level	Lipid selected
DG 34:1	$C_{37}H_{70}O_5$	594.5223	10	Putative	DG(17:0/17:1(9Z)/0:0)[iso2]
DG 36:2	$C_{39}H_{72}O_5$	620.538	8	Putative	DG(16:1(9Z)/20:1(11Z)/0:0)[iso2]
DG O-40:8	$C_{43}H_{70}O_4$	650.5274	4	Putative	1-(8-[3]-ladderane-octanoyl)-2-(8-[1]-ladderane-octanyl)-sn-glycerol
DG 42:6	$C_{45}H_{76}O_5$	696.5693	6	Putative	DG(20:1(11Z)/22:5(7Z,10Z,13Z,16Z,19Z)/0:0)[iso2]
SQDG 16:0_16:0	$C_{41}H_{78}O_{12}S$	794.5214	1	MS/MS	SQDG(16:0/16:0)
Glycerol-2-(9Z,12Z-octadecadienoate)-1-hexadecanoate-3-O-[alpha-D-galactopyranosyl-(1->6)-beta-D-galactopyranoside]	$C_{49}H_{88}O_{15}$	916.6123	1	MS/MS	Glycerol-2-(9Z,12Z-octadecadienoate)-1-hexadecanoate-3-O-[alpha-D-galactopyranosyl-(1->6)-beta-D-galactopyranoside]
Glycerophospholipids					
PG 34:1	$C_{40}H_{77}O_{10}P$	748.5254	17	Putative	PG(19:1(9Z)/15:0)
PG O-36:1	$C_{42}H_{83}O_9P$	762.5775	6	Putative	PG(O-16:0/20:1(11Z))
PG 40:1	$C_{46}H_{89}O_{10}P$	832.6193	8	Putative	PG(18:1(9Z)/22:0)
PG O-38:3	$C_{44}H_{83}O_9P$	786.5775	6	Putative	PG(O-20:0/18:3(6Z,9Z,12Z))
PG 40:6	$C_{46}H_{79}O_{10}P$	822.5411	11	Putative	PG(20:4(5Z,8Z,11Z,14Z)/20:2(11Z,14Z))
LPC O-14:0	$C_{22}H_{48}NO_6P$	453.3219	1	MS/MS	PC(O-14:0/0:0)
PC O-34:5	$C_{42}H_{76}NO_7P$	737.5359	1	MS/MS	PC(P-16:0/18:4(6Z,9Z,12Z,15Z))
PC 36:1	$C_{44}H_{86}NO_8P$	787.6091	26	Putative	PC(16:0/20:1(11Z))
PC O-38:9	$C_{46}H_{76}NO_7P$	785.5359	1	MS/MS	1-(6-[5]-ladderane-hexanoyl)-2-(8-[3]-ladderane-octanyl)-sn-glycerophosphocholine
PC 16:1_16:0	$C_{40}H_{78}NO_8P$	731.5465	2	Putative	PC(16:0/16:1(9Z))
PC 16:0_18:1	$C_{42}H_{82}NO_8P$	759.5778	8	Putative	PC(16:0/18:1(11E))

continued on next page

Supplementary Data 4. (*Continued*)

Compound	Molecular Formula	Molecular weight (DB) g/mol	Isomers	ID Level	Lipid selected
PC 18:1_18:1	$C_{44}H_{84}NO_8P$	785.5935	21	Putative	PC(18:1(15Z)/18:1(15Z))
PC 18:2_16:0	$C_{42}H_{80}NO_8P$	757.5622	12	Putative	PC(16:0/18:2(11Z,13Z))
PC 18:2_18:2	$C_{44}H_{80}NO_8P$	781.5622	5	Putative	PC(18:2(6Z,9Z)/18:2(6Z,9Z))
LPE O-24:0;O	$C_{29}H_{62}NO_7P$	567.4264	1	MS/MS	1-(2-methoxy-tricosanyl)-sn-glycero-3-phosphoethanolamine
PE 36:1	$C_{41}H_{80}NO_8P$	745.5622	17	Putative	PE(16:1(9Z)/20:0)
PE 37:3	$C_{42}H_{78}NO_8P$	755.5465	14	Putative	PE(17:2(9Z,12Z)/20:1(11Z))
PE 38:2	$C_{43}H_{82}NO_8P$	771.5778	14	Putative	PE(20:0/18:2(9Z,12Z))
PE 39:3	$C_{44}H_{82}NO_8P$	783.5778	12	Putative	PE(19:0/20:3(8Z,11Z,14Z))
PE 18:1_18:1	$C_{41}H_{78}NO_8P$	743.5465	3	Putative	PE(18:1(9E)/18:1(9E))
PE 18:2_18:1	$C_{41}H_{76}NO_8P$	741.5309	2	Putative	PE(18:1(9Z)/18:2(9Z,12Z))
PA O-36:0	$C_{39}H_{79}O_7P$	690.5563	3	Putative	PA(O-16:0/20:0)
PE dO-40:0	$C_{45}H_{94}NO_7P$	791.6768	1	MS/MS	beta-hydroarchaetidylethanolamine
PI O-38:1	$C_{47}H_{91}O_{12}P$	878.6248	6	Putative	PI(O-16:0/22:1(11Z))
PI 38:4	$C_{47}H_{83}O_{13}P$	886.5571	13	Putative	PI(18:3(6Z,9Z,12Z)/20:1(11Z))
PI 44:8	$C_{53}H_{87}O_{13}P$	962.5884	3	Putative	PI(22:4(7Z,10Z,13Z,16Z)/22:4(7Z,10Z,13Z,16Z))
PIP 38:2	$C_{47}H_{88}O_{16}P_2$	970.5548	2	Putative	PI(P-18:0/20:2(11Z,14Z))
PIM1 32:1	$C_{47}H_{87}O_{18}P$	970.563	2	Putative	PIM1(16:0/16:1(9Z))
PS 39:2	$C_{45}H_{84}NO_{10}P$	829.5833	12	Putative	PS(19:0/20:2(11Z,14Z))

continued on next page

Supplementary Data 4. (*Continued*)

Compound	Molecular Formula	Molecular weight (DB) g/mol	Isomers	ID Level	Lipid selected
PS 39:3	$C_{45}H_{82}NO_{10}P$	827.5676	12	Putative	PS(22:1(11Z)/17:2(9Z,12Z))
PS 36:0	$C_{42}H_{82}NO_{10}P$	791.5676	8	Putative	PS(20:0/16:0)
PS 39:4	$C_{45}H_{80}NO_{10}P$	825.552	10	Putative	PS(17:0/22:4(7Z,10Z,13Z,16Z))
PS(DiMe(11,3)/DiMe(13,5))	$C_{50}H_{84}NO_{12}P$	921.5731	1	MS/MS	PS(DiMe(11,3)/DiMe(13,5))
Polyketides					
Anacardic acid	$C_{22}H_{30}O_3$	342.2195	1	MS/MS	Anacardic acid
Prenol lipids					
Lucidenic acid	$C_{27}H_{38}O_7$	474.2618	3	Putative	Lucidenic acid B
Hydroxyglabrolide	$C_{30}H_{44}O_5$	484.3189	1	MS/MS	24-Hydroxyglabrolide
Zeaxanthin diglucoside/ Zeaxanthin beta-D-diglucoside	$C_{52}H_{76}O_{12}$	892.5337	1	MS/MS	Zeaxanthin diglucoside
tocopheronolactone	$C_{16}H_{22}O_4$	278.1518	1	MS/MS	alpha-tocopheronolactone
tocotrienol	$C_{28}H_{42}O_2$	410.3185	1	MS/MS	gamma-tocotrienol
Flavoxanthin	$C_{40}H_{56}O_3$	584.4229	1	MS/MS	Flavoxanthin
Triterpenoid	$C_{30}H_{48}O_7S$	552.3121	1	MS/MS	Triterpenoid
Curcumadiol	$C_{15}H_{26}O_2$	238.1933	1	MS/MS	Curcumadiol
Cedryl acetate	$C_{17}H_{28}O_2$	264.2089	1	MS/MS	Cedryl acetate
Armillane	$C_{23}H_{32}O_7$	420.2148	1	MS/MS	Armillane
Sphingolipids					
Termitomycesphin A	$C_{41}H_{77}NO_{10}$	743.5548	1	MS/MS	Termitomycesphin A
CerPE 40:3;O3	$C_{42}H_{81}N_2O_7P$	756.5781	1	MS/MS	CerPE(d14:2(4E,6E)/24:1(15Z)(2OH))
CerP 44:1;O2	$C_{44}H_{88}NO_6P$	757.6349	1	MS/MS	CerP(d18:1/26:0)
HexCer 37:0;O4	$C_{43}H_{85}NO_{10}$	775.6173	1	MS/MS	PI-Cer(d18:0/26:0)
HexCer 36:1;O4	$C_{42}H_{81}NO_{10}$	759.586	1	MS/MS	GlcCer(t18:1(8Z)/18:0(2OH[S]))
HexCer 39:2;O2	$C_{45}H_{85}NO_8$	767.6275	1	MS/MS	GalCer(d18:2/21:0)

continued on next page

Supplementary Data 4. (*Continued*)

Compound	Molecular Formula	Molecular weight (DB) g/mol	Isomers	ID Level	Lipid selected
HexCer 40:2;O3	$C_{46}H_{87}NO_9$	797.6381	1	MS/MS	GlcCer(d18:2(4E,8Z)/22:0(2OH[R]))
N,N-dimethyl-Safingol	$C_{20}H_{43}NO_2$	329.3294	1	MS/MS	N,N-dimethyl-Safingol
SPB 17:0;O2	$C_{17}H_{37}NO_2$	287.2824	1	MS/MS	C17 Sphinganine
Cer 40:0;O3	$C_{40}H_{81}NO_4$	639.6166	2	Putative	Cer(t18:0/22:0)
Cer 40:0;O4	$C_{40}H_{81}NO_5$	655.6115	1	MS/MS	Cer(t16:0(15Me)/23:0(2OH[R]))
Cer 41:0;O4	$C_{41}H_{83}NO_5$	669.6271	1	MS/MS	Cer(t17:0/24:0(2OH))
Cer 44:0;O4	$C_{44}H_{89}NO_5$	711.6741	1	MS/MS	Cer(t18:0/26:0(2OH))
Cer 44:0;O3	$C_{44}H_{89}NO_4$	695.6792	2	Putative	Cer(d18:0/26:0(2OH))
CerPE 38:3;O3	$C_{40}H_{77}N_2O_7P$	728.5468	2	Putative	CerPE(d14:2(4E,6E)/24:1(15Z)(2OH))
Steroids and steroid derivatives					
Benzoyloxystigmast-5-ene-3-ol	$C_{36}H_{54}O_3$	534.4073	1	MS/MS	(3b,24R,25x)-26-Benzoyloxystigmast-5-ene-3-ol
Dehydroteasterone	$C_{28}H_{46}O_4$	446.3396	1	MS/MS	3-Dehydroteasterone
Torvonin A	$C_{39}H_{64}O_{12}$	724.4398	1	MS/MS	Torvonin A
Sterol Lipids					
ST 29:2;O2	$C_{29}H_{48}O_2$	428.3654	1	MS/MS	Sarcophytosterol
Tetrapyrroles and derivatives					
Chlorophyll b	$C_{55}H_{71}MgN_4O_6$	907.5219	1	MS/MS	Chlorophyll b
