# Supplementary material for: Predicting the microalgae lipid profile obtained by supercritical fluid extraction using a machine learning model
Source: Front Chem. 2024 Oct 25;12:1480887. doi: 10.3389/fchem.2024.1480887 (PMC11543471; doi:10.3389/fchem.2024.1480887)
Supplement: Supplementary file 2 [file DataSheet6.docx]

**Supplementary Data 6**


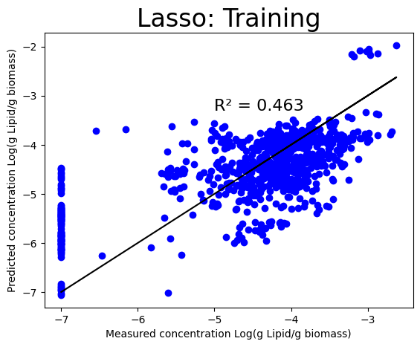

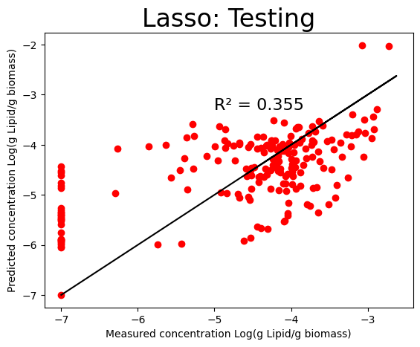

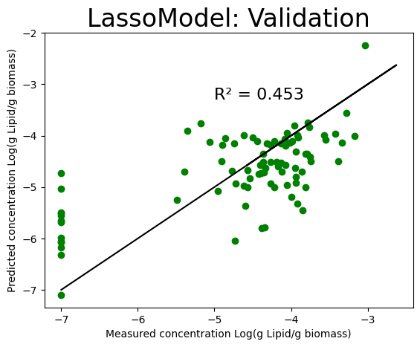


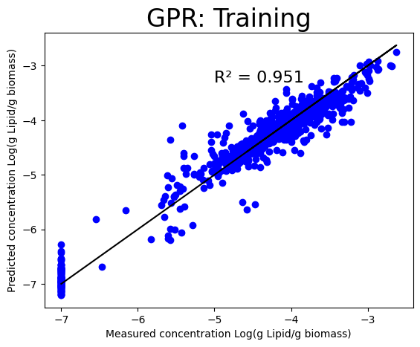

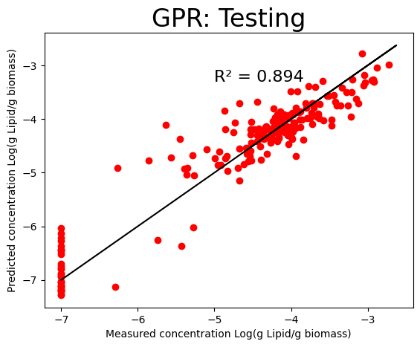

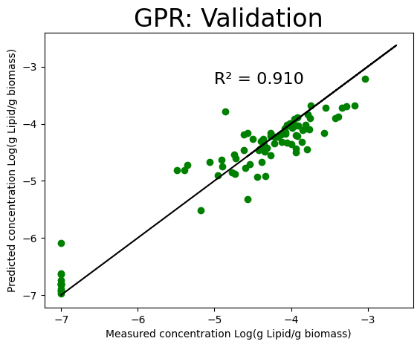


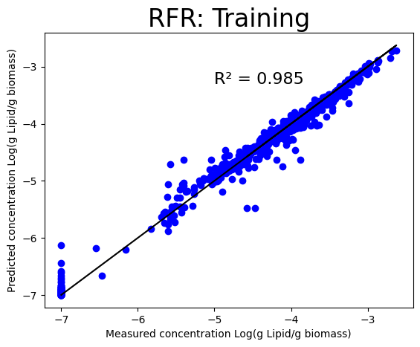

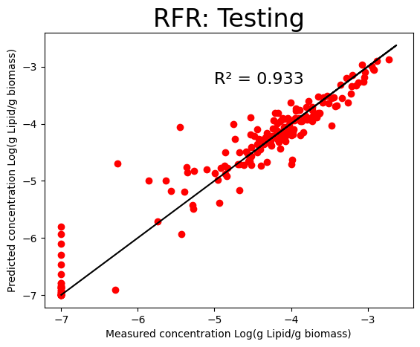

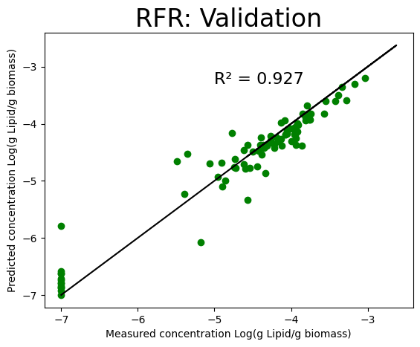


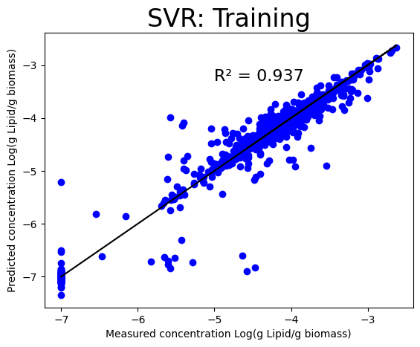

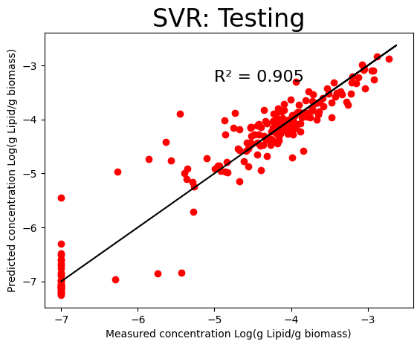

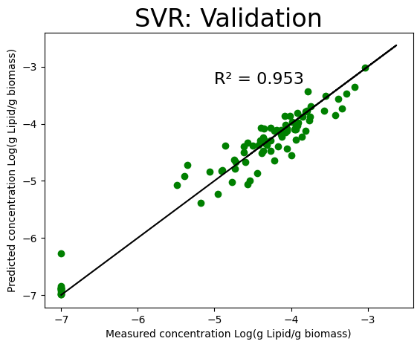


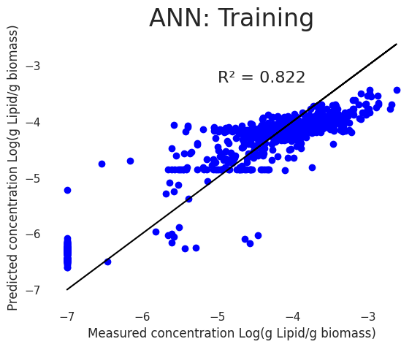

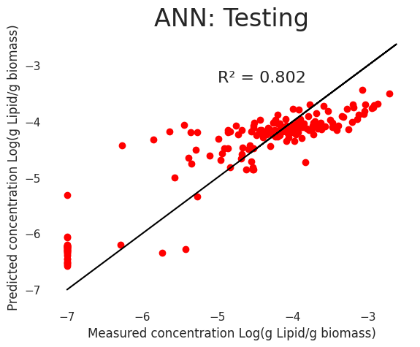

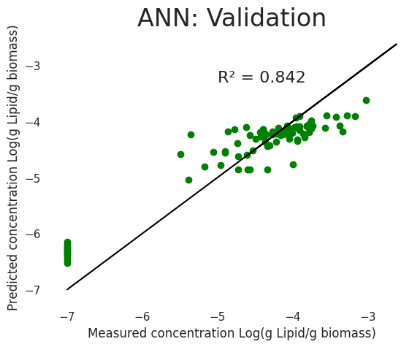


***Figure SD6.*** Regression results for the regression models using training, test data, validation data from SC5
