# Supplementary material for: Predicting the microalgae lipid profile obtained by supercritical fluid extraction using a machine learning model
Source: Front Chem. 2024 Oct 25;12:1480887. doi: 10.3389/fchem.2024.1480887 (PMC11543471; doi:10.3389/fchem.2024.1480887)
Supplement: Supplementary file 3 [file DataSheet5.docx]

**Supplementary Data 5**


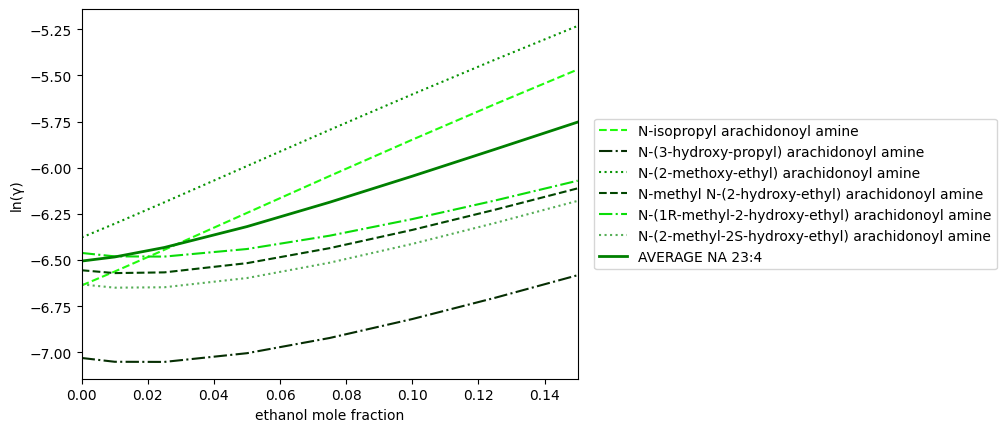


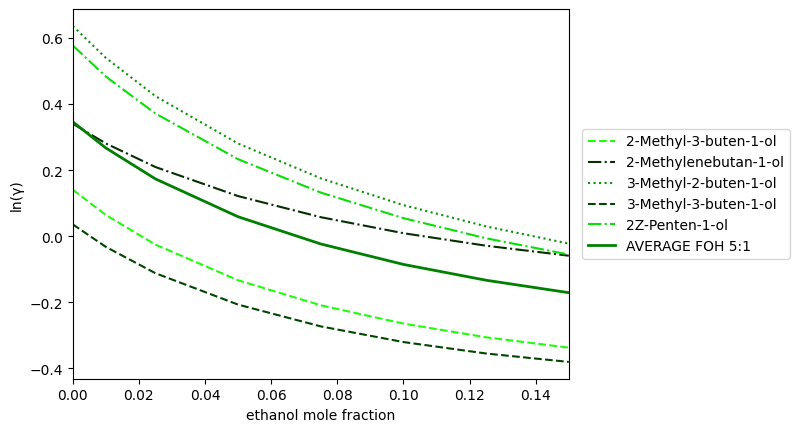


***Figure SD5.*** IDAC calculations for NA 23:4 (a), FOH 5:1 (b) candidates at 50 °C as a function of the mole fraction of ethanol.
