# Supplementary material for: Predicting the microalgae lipid profile obtained by supercritical fluid extraction using a machine learning model
Source: Front Chem. 2024 Oct 25;12:1480887. doi: 10.3389/fchem.2024.1480887 (PMC11543471; doi:10.3389/fchem.2024.1480887)
Supplement: Supplementary file 4 [file DataSheet3.pdf]

Supplementary Data 3

Lipids identified during supercritical fluid extraction. Lipids were identified by metabolic fingerprinting using GL-RP-LC-ESI (+/-)-QTOF-MS

									Relative Abundance (%)											
**Compound**	**Formula**	**Mass**	**RT (min)**	**Mass Error (ppm)**	**Adduct**	**Analytical platform**	**Detection Mode**	**ID level**	**SC1**	**SC2**	**SC3**	**SC4**	**SC5**	**SC6**	**SC7**	**SC8**	**SC9**	**SC10**	**SC11**	**SC12**
Carbohydrates and carbohydrate conjugates																				
Armillarin	$C_{24}H_{30}O_6$	414.2042	1.34	4	[M+H]$^+$	GL-RP-LC-QTOF-MS	ESI +	3	1.38	1.00	1.16	1.03	0.80	0.71	1.02	1.00	0.81	0.95	0.77	0.80
Fatty Acyls																				
aminopentanoic acid	$C_5H_{11}NO_2$	117.079	0.67	4	[M+Na]$^+$	GL-RP-LC-QTOF-MS	ESI +	3	0.57	0.22	0.26	0.16	0.20	0.19	0.20	0.18	0.17	0.18	0.17	0.19
FAL 16:0	$C_{16}H_{32}O$	240.2453	2.3	5	[M+NH$_4$]$^+$	GL-RP-LC-QTOF-MS	ESI +	3	0.17	0.17	0.09	0.35	0.18	0.11	0.10	1.00	0.05	0.26	1.98	0.56
FA 22:5;O	$C_{22}H_{34}O_3$	346.2508	4.91	0	[M-H]$^-$	GL-RP-LC-QTOF-MS	ESI -	3	2.88	0.89	2.03	0.72	1.89	1.30	1.35	0.74	1.41	0.68	0.60	1.67
FA 22:6;O	$C_{22}H_{32}O_3$	344.2351	4.41	0	[M-H]$^-$	GL-RP-LC-QTOF-MS	ESI -	3	1.91	0.42	1.16	0.31	1.24	0.71	0.80	0.39	0.80	0.36	0.31	1.04
FA 22:4;O	$C_{22}H_{36}O_3$	348.2664	5.62	0	[M-H]$^-$	GL-RP-LC-QTOF-MS	ESI -	2	0.54	0.11	0.28	0.08	0.27	0.16	0.14	0.13	0.13	0.06	0.06	0.18
FA 26:1;O2	$C_{26}H_{50}O_4$	426.3709	7.08	4	[M+H]$^+$	GL-RP-LC-QTOF-MS	ESI +	3	0.35	0.27	0.31	0.42	1.08	0.19	0.10	0.07	0.14	0.11	0.65	0.09
FOH 5:1	$C_5H_{10}O$	86.0732	0.67	8	[M+NH$_4$]$^+$	GL-RP-LC-QTOF-MS	ESI +	3	0.11	0.38	0.49	0.17	0.03	0.50	0.10	0.14	0.04	0.28	0.05	0.59
Stearamide	$C_{18}H_{37}NO$	283.2875	5.11	5	[M+H]$^+$	GL-RP-LC-QTOF-MS	ESI +	3	0.41	0.00	0.00	0.00	0.00	0.00	0.00	0.00	0.00	0.00	0.00	0.00
NA 23:4	$C_{23}H_{39}NO$	345.3032	5.19	5	[M+H]$^+$	GL-RP-LC-QTOF-MS	ESI +	3	0.06	0.00	0.00	0.00	0.00	0.00	0.00	0.00	0.00	0.00	0.00	0.00
NA 25:4	$C_{25}H_{43}NO$	373.3345	6.09	4	[M+H]$^+$	GL-RP-LC-QTOF-MS	ESI +	3	0.05	0.00	0.00	0.00	0.00	0.00	0.00	0.00	0.00	0.00	0.00	0.00
6E-Heneicosen-11-one	$C_{21}H_{40}O$	308.3079	5.61	6	[M+NH$_4$]$^+$	GL-RP-LC-QTOF-MS	ESI +	3	0.03	0.00	0.00	0.00	0.00	0.00	0.00	0.00	0.00	0.00	0.00	0.00
12-Nonadecen-9-one	$C_{19}H_{36}O$	280.2766	5.36	5	[M+NH$_4$]$^+$	GL-RP-LC-QTOF-MS	ESI +	3	0.02	0.00	0.00	0.00	0.00	0.00	0.00	0.00	0.00	0.00	0.00	0.00
WE 24:2;O4	$C_{24}H_{44}O_6$	428.3138	4.38	0	[M+Cl]$^-$	GL-RP-LC-QTOF-MS	ESI -	3	0.00	0.00	0.00	0.00	0.00	0.00	0.00	0.00	0.00	0.00	0.18	0.00
Glycerolipids																				
MGDG 16:0_18:1	$C_{43}H_{80}O_{10}$	756.5752	9.35	2	[M+HCOOH-H]$^-$	GL-RP-LC-QTOF-MS	ESI -	2	0.99	1.30	1.33	1.31	1.20	1.09	1.18	1.17	0.47	0.40	0.49	0.41
MGDG 16:0_18:2	$C_{43}H_{78}O_{10}$	754.5595	8.71	2	[M+NH4]+/M+HCOOH-H	GL-RP-LC-QTOF-MS	ESI +/ESI	2	3.38	4.23	3.91	3.98	4.45	3.69	3.64	4.06	3.15	2.66	3.03	2.62
MGDG 18:2_18:2	$C_{45}H_{78}O_{10}$	778.5595	8.15	2	M+HCOOH-H/M+Na	GL-RP-LC-QTOF-MS	ESI -/ESI +	2	1.35	1.72	1.70	1.64	1.73	1.86	1.49	1.68	2.30	2.14	2.43	2.02
MGDG 18:1_18:1	$C_{45}H_{82}O_{10}$	782.5908	9.45	1	M+HCOOH-H/M+NH4	GL-RP-LC-QTOF-MS	ESI -/ESI +	2	0.46	0.71	0.81	0.85	0.58	0.71	0.75	0.77	0.38	0.33	0.39	0.33
MGDG 36:6	$C_{45}H_{74}O_{10}$	774.5282	7.84	6	[M+HCOOH-H]$^-$	GL-RP-LC-QTOF-MS	ESI -	3	0.01	0.03	0.00	0.10	0.07	0.07	0.54	0.49	0.06	0.05	0.08	0.07
DG 34:1	$C_{37}H_{70}O_5$	594.5223	10.56	5	[M+H]$^+$	GL-RP-LC-QTOF-MS	ESI +	3	0.66	0.61	0.85	0.63	0.73	0.47	0.30	0.32	0.28	0.20	0.22	0.22
DG 36:2	$C_{39}H_{72}O_5$	620.538	10.62	5	[M+H]$^+$	GL-RP-LC-QTOF-MS	ESI +	3	1.01	0.95	1.28	0.97	0.95	0.76	0.44	0.47	0.47	0.32	0.35	0.36
DG O-40:8	$C_{43}H_{70}O_4$	650.5274	9.28	8	[M-H]$^-$	GL-RP-LC-QTOF-MS	ESI -	3	0.47	0.49	0.43	0.39	0.47	0.35	0.26	0.29	0.35	0.62	0.59	0.45
DG 42:6	$C_{45}H_{76}O_5$	696.5693	10.11	6	[M-H-H$_2$O]$^-$	GL-RP-LC-QTOF-MS	ESI -	3	0.78	0.88	0.82	0.81	0.91	0.67	0.52	0.51	0.59	0.98	0.98	0.72
SQDG 16:0_16:0	$C_{41}H_{78}O_{12}S$	794.5214	7.75	1	[M-H]$^-$	GL-RP-LC-QTOF-MS	ESI -	2	0.02	0.04	0.01	0.14	0.09	0.12	0.72	0.61	0.26	0.16	0.29	0.28
Glycerol 2-(9Z,12Z-octadecadienoate) 1-hexadecanoate 3-O-[alpha-D-galactopyranosyl-(1->6)-beta-D-galactopyranoside]	$C_{49}H_{88}O_{15}$	916.6123	8.05	2	M-H/M+NH4	GL-RP-LC-QTOF-MS	ESI -/ESI +	3	0.30	0.81	0.33	1.07	1.23	1.03	2.72	2.53	0.95	0.71	0.88	0.80
Glycerophospholipids																				
PG 34:1	$C_{40}H_{77}O_{10}P$	748.5254	8.22	2	[M-H]$^-$	GL-RP-LC-QTOF-MS	ESI -	3	0.05	0.13	0.03	0.19	0.15	0.17	0.46	0.54	0.11	0.09	0.11	0.09
PG O-36:1	$C_{42}H_{83}O_9P$	762.5775	7.98	4	[M+Cl]$^-$	GL-RP-LC-QTOF-MS	ESI -	4	0.48	0.61	0.58	0.57	0.65	0.42	0.51	0.59	0.24	0.14	0.16	0.13
PG 40:1	$C_{46}H_{89}O_{10}P$	832.6193	11.01	8	[M+K]$^+$	GL-RP-LC-QTOF-MS	ESI +	3	7.26	4.14	6.72	3.66	4.94	3.89	2.32	2.01	1.22	0.82	3.43	1.64
PG O-38:3	$C_{44}H_{83}O_9P$	786.5775	7.57	4	[M+Cl]$^-$	GL-RP-LC-QTOF-MS	ESI -	4	0.21	0.32	0.32	0.33	0.29	0.63	0.30	0.32	1.01	0.98	1.11	0.93
PG 40:6	$C_{46}H_{79}O_{10}P$	822.5411	8.7	9	[M+HCOOH-H]$^-$	GL-RP-LC-QTOF-MS	ESI -	4	0.77	0.89	0.90	0.82	0.95	0.69	0.82	0.89	0.59	0.46	0.52	0.47
LPC O-14:0	$C_{22}H_{48}NO_6P$	453.3219	6.04	6	M+Na	GL-RP-LC-QTOF-MS	ESI +	4	0.03	0.00	0.00	0.00	0.00	0.00	0.00	0.00	0.00	0.00	0.00	0.00
PC O-34:5	$C_{42}H_{76}NO_7P$	737.5359	8.85	1	[M+NH$_4$]$^+$	GL-RP-LC-QTOF-MS	ESI +	3	1.37	1.59	1.51	1.60	1.86	1.40	1.55	1.46	0.82	0.73	0.79	0.83
PC 36:1	$C_{44}H_{86}NO_8P$	787.6091	9.83	5	[M+HCOOH-H]-/[M+H]$^+$	GL-RP-LC-QTOF-MS	ESI -/ESI +	3	0.75	1.30	0.79	1.76	1.31	1.71	1.90	1.68	1.22	1.46	1.51	1.60
PC O-38:9	$C_{46}H_{76}NO_7P$	785.5359	8.91	2	[M+NH$_4$]$^+$	GL-RP-LC-QTOF-MS	ESI +	3	0.43	0.42	0.59	0.39	0.39	0.22	0.28	0.26	0.15	0.11	0.10	0.13
PC 16:1_16:0	$C_{40}H_{78}NO_8P$	731.5465	8.15	5	[M+HCOOH-H]-/[M+H]$^+$	GL-RP-LC-QTOF-MS	ESI -/ESI +	2	0.63	1.00	0.58	1.12	1.18	1.65	1.74	1.73	1.46	1.60	1.65	1.70
PC 16:0_18:1	$C_{42}H_{82}NO_8P$	759.5778	8.8	2	[M+HCOOH-H]$^-$	GL-RP-LC-QTOF-MS	ESI -	2	3.30	4.69	3.48	4.75	4.05	5.00	4.95	5.52	6.38	6.38	6.82	6.08
PC 18:1_18:1	$C_{44}H_{84}NO_8P$	785.5935	9.06	5	M+HCOOH-H/M+H	GL-RP-LC-QTOF-MS	ESI -/ESI +	2	6.05	7.97	6.54	8.54	7.24	7.98	9.45	9.45	7.79	7.97	7.78	9.01
PC 18:2_16:0	$C_{42}H_{80}NO_8P$	757.5622	8.32	3	M+H/M+HCOOH-H	GL-RP-LC-QTOF-MS	ESI -/ESI +	2	3.86	5.35	2.96	5.05	5.62	7.18	6.19	6.16	7.78	8.12	8.15	9.09
PC 18:2_18:2	$C_{44}H_{80}NO_8P$	781.5622	8.97	2	[M+HCOOH-H]-/[M+H]$^+$	GL-RP-LC-QTOF-MS	ESI -/ESI +	2	1.15	1.39	1.18	1.45	1.32	1.32	1.50	1.50	1.40	1.42	1.37	1.65
LPE O-24:0;O	$C_{29}H_{62}NO_7P$	567.4264	6.13	4	[M+H]$^+$	GL-RP-LC-QTOF-MS	ESI +	3	1.29	1.87	1.68	1.71	1.65	1.53	1.91	1.62	1.04	0.95	0.80	0.87
PE 36:1	$C_{41}H_{80}NO_8P$	745.5622	8.55	8	[M+H]$^+$	GL-RP-LC-QTOF-MS	ESI +	3	0.70	1.24	0.94	1.74	1.12	2.57	2.03	2.02	2.83	3.21	3.23	3.50

continued on next page

Supplementary Data 3. (*Continued*)

Compound	Formula	Mass	RT (min)	Mass Error (ppm)	Adduct	Analytical platform	Detection Mode	ID level	SC1	SC2	SC3	SC4	SC5	SC6	SC7	SC8	SC9	SC10	SC11	SC12
									Relative Abundance (%)											
PE 37:3	$C_{42}H_{78}NO_8P$	755.5465	7.72	4	$[M+H]^+$	GL-RP-LC-QTOF-MS	ESI +		0.07	0.10	0.06	0.13	0.05	0.35	0.07	0.06	0.71	0.91	0.94	0.90
PE 38:2	$C_{43}H_{82}NO_8P$	771.5778	8.65	5	$[M+H]^+$	GL-RP-LC-QTOF-MS	ESI +	3	0.11	0.28	0.19	0.22	0.26	0.50	0.49	0.48	0.44	0.54	0.59	0.57
PE 39:3	$C_{44}H_{82}NO_8P$	783.5778	8.39	5	$[M+H]^+$	GL-RP-LC-QTOF-MS	ESI +	3	1.79	2.89	1.49	2.73	2.87	4.51	3.43	3.49	5.17	5.54	5.47	6.16
PE 18:1_18:1	$C_{41}H_{78}NO_8P$	743.5465	9.05	2	$[M-H]^-$	GL-RP-LC-QTOF-MS	ESI -	2	0.57	0.89	0.37	1.27	1.23	1.51	2.25	2.80	2.78	2.70	2.97	2.63
PE 18:2_18:1	$C_{41}H_{76}NO_8P$	741.5309	8.41	1	$[M-H]^-$	GL-RP-LC-QTOF-MS	ESI -	2	0.07	0.10	0.04	0.11	0.13	0.21	0.20	0.26	0.58	0.58	0.63	0.55
PA O-36:0	$C_{39}H_{79}O_7P$	690.5563	11.7	9	$[M+NH_4]^+$	GL-RP-LC-QTOF-MS	ESI +	3	0.48	0.37	0.38	0.22	0.46	0.26	0.09	0.19	0.84	0.87	0.51	1.01
PE dO-40:0	$C_{45}H_{94}NO_7P$	791.6768	11.7	9	$[M+H]^+$	GL-RP-LC-QTOF-MS	ESI +	3	0.46	0.36	0.37	0.23	0.43	0.24	0.10	0.20	0.75	0.74	0.48	0.86
PI O-38:1	$C_{47}H_{91}O_{12}P$	878.6248	7.47	4	$[M+Cl]^-$	GL-RP-LC-QTOF-MS	ESI -	3	0.05	0.14	0.07	0.23	0.20	0.24	0.59	0.56	0.28	0.22	0.27	0.25
PI 38:4	$C_{47}H_{83}O_{13}P$	886.5571	10.62	7	$[M+H]^+$	GL-RP-LC-QTOF-MS	ESI +	3	0.26	0.24	0.59	0.09	0.13	0.13	0.06	0.04	0.05	0.04	0.12	0.07
PI 44:8	$C_{53}H_{87}O_{13}P$	962.5884	9.56	3	$[M+Cl]^-$	GL-RP-LC-QTOF-MS	ESI -	3	1.20	3.30	1.53	0.33	0.31	0.12	0.13	0.14	0.13	0.06	0.06	0.06
PIP 38:2	$C_{47}H_{88}O_{16}P_2$	970.5548	9.36	2	$[M+Cl]^-$	GL-RP-LC-QTOF-MS	ESI -	4	1.51	1.83	1.93	2.13	1.95	1.73	1.81	2.16	1.77	1.37	1.36	1.31
PIM1 32:1	$C_{47}H_{87}O_{18}P$	970.563	9.73	3	$[M+H-H_2O]^+$	GL-RP-LC-QTOF-MS	ESI +	3	0.45	1.00	0.51	0.12	0.12	0.05	0.06	0.05	0.04	0.02	0.02	0.02
PS 39:2	$C_{45}H_{84}NO_{10}P$	829.5833	8.22	2	$[M-H]^-$	GL-RP-LC-QTOF-MS	ESI -	3	0.51	0.76	0.45	0.64	0.64	1.09	0.72	0.84	1.82	1.91	2.09	1.79
PS 39:3	$C_{45}H_{82}NO_{10}P$	827.5676	8.78	1	$[M+HCOOH-H]^-$	GL-RP-LC-QTOF-MS	ESI -	3	0.44	0.59	0.51	0.57	0.52	0.54	0.57	0.64	0.77	0.74	0.78	0.73
PS 36:0	$C_{42}H_{82}NO_{10}P$	791.5676	8.38	2	$[M-H]^-$	GL-RP-LC-QTOF-MS	ESI -	3	0.25	0.46	0.32	0.56	0.34	0.74	0.58	0.66	1.11	1.22	1.32	1.13
PS 39:4	$C_{45}H_{80}NO_{10}P$	825.552	8.14	1	$[M+HCOOH-H]^-$	GL-RP-LC-QTOF-MS	ESI -	3	0.17	0.21	0.15	0.19	0.20	0.29	0.18	0.22	0.47	0.48	0.52	0.45
PS(DiMe(11,3)/DiMe(13,5))	$C_{50}H_{84}NO_{12}P$	921.5731	8.17	2	$[M+NH_4]^+$	GL-RP-LC-QTOF-MS	ESI +	3	0.04	0.14	0.05	0.22	0.29	0.24	0.86	0.67	0.15	0.12	0.14	0.16
Polyketides																				
Anacardic acid	$C_{22}H_{30}O_3$	342.2195	4.02	0	$[M-H]^-$	GL-RP-LC-QTOF-MS	ESI -	3	2.29	0.65	1.55	0.50	1.65	1.06	1.13	0.69	1.16	0.67	0.47	1.52
Prenol lipids																				
Lucidenic acid	$C_{27}H_{38}O_7$	474.2618	4.44	2	$[M+NH_4]^+$	GL-RP-LC-QTOF-MS	ESI +	3	0.03	0.00	0.00	0.00	0.00	0.00	0.00	0.00	0.00	0.00	0.00	0.00
Hydroxyglabrolide	$C_{30}H_{44}O_5$	484.3189	5.34	1	$[M+H]^+$	GL-RP-LC-QTOF-MS	ESI +	3	0.02	0.00	0.00	0.00	0.00	0.00	0.00	0.00	0.00	0.00	0.00	0.00
Zeaxanthin diglucoside/ Zeaxanthin beta-D-diglucoside	$C_{52}H_{76}O_{12}$	892.5337	9.38	3	M+H]+/$[M+HCOOH-H]^-$	GL-RP-LC-QTOF-MS	ESI -/ESI +	3	7.55	9.32	9.72	10.91	9.98	8.67	9.10	10.95	9.65	7.22	7.16	6.87
tocopheronolactone	$C_{16}H_{22}O_4$	278.1518	5.53	4	$[M+H]^+$	GL-RP-LC-QTOF-MS	ESI +	3	0.94	0.07	0.04	0.02	0.04	0.02	0.00	0.00	0.03	0.05	0.01	0.03
tocotrienol	$C_{28}H_{42}O_2$	410.3185	4.89	4	$[M+H]^+$	GL-RP-LC-QTOF-MS	ESI +	3	0.34	0.72	0.56	0.37	0.45	0.27	0.34	0.38	0.44	0.62	0.15	0.58
Flavoxanthin	$C_{40}H_{56}O_3$	584.4229	5.15	5	$[M+H-H_2O]^+$	GL-RP-LC-QTOF-MS	ESI +	4	2.69	2.84	3.14	2.81	3.03	2.53	3.00	2.85	2.10	1.85	1.92	2.22
Triterpenoid	$C_{30}H_{48}O_7S$	552.3121	2.44	8	$[M+H]^+$	GL-RP-LC-QTOF-MS	ESI +	4	0.04	0.00	0.00	0.00	0.00	0.00	0.00	0.00	0.00	0.00	0.00	0.00
Curcumadiol	$C_{15}H_{26}O_2$	238.1933	0.7	0	$[M+Na]^+$	GL-RP-LC-QTOF-MS	ESI +	3	0.96	1.46	1.09	1.42	1.30	1.18	1.69	1.76	1.13	1.11	1.12	1.24
Cedryl acetate	$C_{17}H_{28}O_2$	264.2089	0.71	1	$[M+Na]^+$	GL-RP-LC-QTOF-MS	ESI +	3	0.54	0.61	0.47	0.58	0.58	0.44	0.69	0.69	0.37	0.34	0.37	0.42
Armillane	$C_{23}H_{32}O_7$	420.2148	2.25	6	$[M+H]^+$	GL-RP-LC-QTOF-MS	ESI +	3	0.04	0.00	0.00	0.00	0.00	0.00	0.00	0.00	0.00	0.00	0.00	0.00
Sphingolipids																				
Termitomycesphin A	$C_{41}H_{77}NO_{10}$	743.5548	9.19	6	$[M+H]^+$	GL-RP-LC-QTOF-MS	ESI +	3	0.36	0.60	0.26	0.92	0.89	1.14	1.94	2.08	1.53	1.57	1.63	1.73
CerPE 40:3;O3	$C_{42}H_{81}N_2O_7P$	756.5781	9.49	7	$[M+NH_4]^+$	GL-RP-LC-QTOF-MS	ESI +	3	0.79	1.28	1.27	1.87	1.47	1.60	1.63	1.60	0.80	0.88	0.91	0.96
CerP 44:1;O2	$C_{44}H_{88}NO_6P$	757.6349	8.28	2	$[M+K]^+$	GL-RP-LC-QTOF-MS	ESI +	3	0.56	0.77	0.66	0.80	0.91	0.89	0.74	0.74	0.71	0.66	0.69	0.74
HexCer 37:0;O4	$C_{43}H_{85}NO_{10}$	775.6173	8.93	7	$[M+Na]^+$	GL-RP-LC-QTOF-MS	ESI +	3	0.70	0.91	0.97	1.00	0.95	0.80	0.88	0.85	0.43	0.36	0.39	0.41
HexCer 36:1;O4	$C_{42}H_{81}NO_{10}$	759.586	7.72	3	$[M+Na]^+$	GL-RP-LC-QTOF-MS	ESI +	3	0.19	0.27	0.13	0.22	0.26	0.74	0.27	0.25	1.16	1.42	1.49	1.41
HexCer 39:2;O2	$C_{45}H_{85}NO_8$	767.6275	10.72	1	$[M+HCOOH-H]^-$	GL-RP-LC-QTOF-MS	ESI -	3	1.08	0.98	1.32	1.83	1.53	1.74	1.38	1.43	1.49	1.92	1.58	2.04
HexCer 40:2;O3	$C_{46}H_{87}NO_9$	797.6381	11.27	1	$[M-H]^-$	GL-RP-LC-QTOF-MS	ESI -	3	0.53	0.45	0.76	0.86	0.69	0.80	0.60	0.60	0.64	0.76	0.75	0.77
N,N-dimethyl-Safingol	$C_{20}H_{43}NO_2$	329.3294	6.05	5	$[M+H-H_2O]^+$	GL-RP-LC-QTOF-MS	ESI +	3	0.28	0.00	0.00	0.00	0.00	0.00	0.00	0.00	0.00	0.00	0.00	0.00
SPB 17:0;O2	$C_{17}H_{37}NO_2$	287.2824	4.49	4	$[M+H-H_2O]^+$	GL-RP-LC-QTOF-MS	ESI +	3	0.03	0.00	0.00	0.00	0.00	0.00	0.00	0.00	0.00	0.00	0.00	0.00
Cer 40:0;O3	$C_{40}H_{81}NO_4$	639.6166	10.36	2	$[M+HCOOH-H]^-$	GL-RP-LC-QTOF-MS	ESI -	3	0.27	0.27	0.46	0.65	0.40	0.61	0.51	0.51	0.55	0.68	0.59	0.74
Cer 40:0;O4	$C_{40}H_{81}NO_5$	655.6115	10.58	3	$[M-H]^-$	GL-RP-LC-QTOF-MS	ESI -	3	0.76	0.74	1.18	1.36	1.04	1.22	0.89	0.90	1.16	1.26	1.28	1.30
Cer 41:0;O4	$C_{41}H_{83}NO_5$	669.6271	11.48	1	$[M-H]^-$	GL-RP-LC-QTOF-MS	ESI -	3	0.65	0.50	0.73	0.67	0.79	0.53	0.36	0.34	0.48	0.47	0.49	0.46
Cer 44:0;O4	$C_{44}H_{89}NO_5$	711.6741	11.89	2	$[M-H]^-$	GL-RP-LC-QTOF-MS	ESI -	3	0.45	0.37	0.65	0.72	0.58	0.61	0.56	0.47	0.56	0.64	0.70	0.69
Cer 44:0;O3	$C_{44}H_{89}NO_4$	695.6792	11.7	2	$[M+HCOOH-H]^-$	GL-RP-LC-QTOF-MS	ESI -	3	0.33	0.30	0.37	0.54	0.46	0.46	0.47	0.50	0.55	0.69	0.54	0.72
CerPE 38:3;O3	$C_{40}H_{77}N_2O_7P$	728.5468	8.55	3	$[M+HCOOH-H]^-$	GL-RP-LC-QTOF-MS	ESI -	3	0.52	0.70	0.65	0.65	0.82	0.45	0.54	0.63	0.11	0.02	0.03	0.02
Steroids and steroid derivatives																				
Benzoyloxystigmast-5-ene-3-ol	$C_{36}H_{54}O_3$	534.4073	9.05	10	$[M+NH_4]^+$	GL-RP-LC-QTOF-MS	ESI +	3	2.08	1.67	3.07	1.52	1.77	0.92	1.12	1.01	0.84	0.51	0.61	0.53

continued on next page

Supplementary Data 3. *(Continued)*

Compound	Formula	Mass	RT (min)	Mass Error (ppm)	Adduct	Analytical platform	Detection Mode	ID level	Relative Abundance (%)											
									SC1	SC2	SC3	SC4	SC5	SC6	SC7	SC8	SC9	SC10	SC11	SC12
Dehydroteasterone	$C_{28}H_{46}O_4$	446.3396	5.45	4	$[M+H-H_2O]^+$	GL-RP-LC-QTOF-MS	ESI +	3	0.48	0.52	0.56	0.40	0.49	0.34	0.25	0.38	0.49	0.47	0.28	0.53
Torvonin A	$C_{39}H_{64}O_{12}$	724.4398	4.02	9	$[M-H-H_2O]^-$	GL-RP-LC-QTOF-MS	ESI -	4	1.04	0.04	0.29	0.03	0.50	0.16	0.18	0.05	0.14	0.05	0.02	0.32
Sterol Lipids																				
ST 29:2;O2	$C_{29}H_{48}O_2$	428.3654	7.46	5	$[M+H]^+$	GL-RP-LC-QTOF-MS	ESI +	3	0.46	0.68	0.85	0.85	0.43	0.42	0.22	0.49	1.08	0.58	0.34	0.69
Tetrapyrroles and derivatives																				
Chlorophyll b	$C_{55}H_{71}MgN_4O_6$	907.5219	9.25	4	$[M+H]^+$	GL-RP-LC-QTOF-MS	ESI +	3	0.47	0.85	1.27	0.19	0.24	0.17	0.13	0.04	0.04	0.03	0.04	0.02
Unknown			0.69			GL-RP-LC-QTOF-MS	ESI +		1.54	2.03	1.62	1.89	1.75	1.84	2.44	2.57	1.64	1.76	1.78	1.95
Unknown			10.42			GL-RP-LC-QTOF-MS	ESI +		0.99	0.96	1.37	0.84	0.96	0.57	0.55	1.08	0.90	0.65	0.41	0.74
Unknown			0.65			GL-RP-LC-QTOF-MS	ESI +		0.62	0.65	0.61	0.62	0.57	0.55	0.66	0.69	0.44	0.45	0.48	0.61
Unknown			0.72			GL-RP-LC-QTOF-MS	ESI +		0.60	0.63	0.58	0.57	0.59	0.47	0.64	0.65	0.39	0.37	0.39	0.50
Unknown			9.36			GL-RP-LC-QTOF-MS	ESI +		0.68	0.78	0.96	0.59	0.82	0.42	0.56	0.61	0.55	0.30	0.38	0.38
Unknown			11.16			GL-RP-LC-QTOF-MS	ESI +		0.56	0.57	0.70	0.37	0.53	0.25	0.34	0.32	0.31	0.17	0.19	0.17
Unknown			5.53			GL-RP-LC-QTOF-MS	ESI +		1.79	0.16	0.14	0.05	0.13	0.05	0.00	0.00	0.11	0.11	0.06	0.05
Unknown			1.26			GL-RP-LC-QTOF-MS	ESI +		1.22	0.12	0.09	0.11	0.03	0.34	0.04	0.01	0.09	0.02	0.00	0.04
Unknown			2.55			GL-RP-LC-QTOF-MS	ESI +		0.58	0.07	0.05	0.05	0.04	0.13	0.02	0.01	0.04	0.01	0.03	0.02
Unknown			1.19			GL-RP-LC-QTOF-MS	ESI +		0.58	0.10	0.04	0.06	0.07	0.13	0.02	0.01	0.04	0.01	0.01	0.02
Unknown			1.93			GL-RP-LC-QTOF-MS	ESI +		0.57	0.06	0.04	0.05	0.01	0.13	0.02	0.00	0.04	0.01	0.00	0.02
Unknown			1.8			GL-RP-LC-QTOF-MS	ESI +		0.59	0.07	0.02	0.03	0.04	0.08	0.01	0.00	0.02	0.01	0.00	0.01
Unknown			5.53			GL-RP-LC-QTOF-MS	ESI +		0.57	0.00	0.00	0.00	0.00	0.00	0.00	0.00	0.00	0.00	0.00	0.00
Unknown			8.97			GL-RP-LC-QTOF-MS	ESI +		0.18	0.63	0.19	0.81	0.65	0.81	0.90	0.88	0.73	0.78	0.78	0.89
Unknown			1.46			GL-RP-LC-QTOF-MS	ESI +		0.33	0.32	0.15	0.62	0.42	0.23	0.21	1.81	0.10	0.58	2.13	1.20
Unknown			7.91			GL-RP-LC-QTOF-MS	ESI +		0.12	0.18	0.16	0.18	0.19	0.58	0.18	0.16	0.79	0.88	0.98	0.91
Unknown			0.78			GL-RP-LC-QTOF-MS	ESI +		0.07	0.00	0.05	0.04	0.00	0.00	0.14	0.00	2.69	2.39	1.74	0.23
Unknown			0.78			GL-RP-LC-QTOF-MS	ESI +		0.07	0.00	0.05	0.04	0.00	0.00	0.14	0.00	2.79	2.47	1.77	0.22
Unknown			0.79			GL-RP-LC-QTOF-MS	ESI +		0.04	0.00	0.03	0.03	0.00	0.00	0.07	0.00	1.61	1.43	0.99	0.12
Unknown			0.78			GL-RP-LC-QTOF-MS	ESI +		0.00	0.00	0.00	0.00	0.00	0.00	0.00	0.00	1.41	1.23	0.87	0.10
Unknown			0.79			GL-RP-LC-QTOF-MS	ESI +		0.00	0.00	0.00	0.00	0.00	0.00	0.03	0.00	0.59	0.52	0.34	0.04
Unknown			0.85			GL-RP-LC-QTOF-MS	ESI +		0.02	0.01	0.01	0.01	0.01	0.01	0.00	0.00	0.01	1.10	0.03	0.25
Unknown			8.92			GL-RP-LC-QTOF-MS	ESI +		0.44	0.52	0.54	0.54	0.54	0.44	0.49	0.49	0.27	0.23	0.25	0.27
Unknown			8.46			GL-RP-LC-QTOF-MS	ESI +		0.29	0.11	0.53	0.22	0.36	0.14	0.19	0.23	0.08	0.08	0.08	0.04
Unknown			5.83			GL-RP-LC-QTOF-MS	ESI +		0.29	0.39	0.46	0.72	0.66	0.40	0.57	0.42	0.26	0.17	0.07	0.21
Unknown			5.83			GL-RP-LC-QTOF-MS	ESI +		0.29	0.39	0.46	0.71	0.66	0.40	0.57	0.42	0.26	0.17	0.07	0.21
Unknown			10.86			GL-RP-LC-QTOF-MS	ESI +		0.29	0.23	0.38	0.57	0.46	0.52	0.40	0.36	0.27	0.37	0.28	0.49
Unknown			0.89			GL-RP-LC-QTOF-MS	ESI +		0.03	0.00	0.00	0.00	0.00	0.00	0.00	0.00	0.00	0.00	0.00	0.00
Unknown			3.56			GL-RP-LC-QTOF-MS	ESI +		0.03	0.00	0.00	0.00	0.00	0.00	0.00	0.00	0.00	0.00	0.00	0.00
Unknown			0.9			GL-RP-LC-QTOF-MS	ESI +		0.02	0.00	0.00	0.00	0.00	0.00	0.00	0.00	0.00	0.00	0.00	0.00
Unknown			1.93			GL-RP-LC-QTOF-MS	ESI +		0.02	0.00	0.00	0.00	0.00	0.00	0.00	0.00	0.00	0.00	0.00	0.00
Unknown			11.94			GL-RP-LC-QTOF-MS	ESI +		0.02	0.00	0.00	0.00	0.00	0.00	0.00	0.00	0.00	0.00	0.00	0.00
Unknown			3.59			GL-RP-LC-QTOF-MS	ESI +		0.02	0.00	0.00	0.00	0.00	0.00	0.00	0.00	0.00	0.00	0.00	0.00
Unknown			4.29			GL-RP-LC-QTOF-MS	ESI +		0.02	0.00	0.00	0.00	0.00	0.00	0.00	0.00	0.00	0.00	0.00	0.00
Unknown			1.02			GL-RP-LC-QTOF-MS	ESI +		0.02	0.00	0.00	0.00	0.00	0.00	0.00	0.00	0.00	0.00	0.00	0.00
Unknown			2.67			GL-RP-LC-QTOF-MS	ESI +		0.01	0.00	0.00	0.00	0.00	0.00	0.00	0.00	0.00	0.00	0.00	0.00
Unknown			2.8			GL-RP-LC-QTOF-MS	ESI -		1.83	0.43	0.12	0.18	0.22	0.48	0.09	0.04	0.21	0.06	0.10	0.12
Unknown			4.91			GL-RP-LC-QTOF-MS	ESI -		0.87	0.03	0.23	0.02	0.30	0.10	0.10	0.02	0.08	0.02	0.01	0.14
Unknown			1.46			GL-RP-LC-QTOF-MS	ESI -		0.67	0.09	0.05	0.06	0.10	0.15	0.02	0.03	0.05	0.04	0.06	0.03
Unknown			1.46			GL-RP-LC-QTOF-MS	ESI -		0.73	0.09	0.03	0.06	0.12	0.18	0.02	0.01	0.07	0.01	0.03	0.03
Unknown			0.82			GL-RP-LC-QTOF-MS	ESI -		0.12	0.70	0.16	0.01	0.03	0.01	0.00	0.03	0.04	1.72	0.13	0.75
Unknown			0.82			GL-RP-LC-QTOF-MS	ESI -		0.09	0.47	0.11	0.01	0.02	0.01	0.00	0.02	0.02	1.21	0.08	0.51

continued on next page

Supplementary Data 3. *(Continued)*

Compound	Formula	Mass	RT (min)	Mass Error (ppm)	Adduct	Analytical platform	Detection Mode	ID level	Relative Abundance (%) SC1	SC2	SC3	SC4	SC5	SC6	SC7	SC8	SC9	SC10	SC11	SC12
Unknown			0.82			GL-RP-LC-QTOF-MS	ESI -		0.07	0.29	0.09	0.00	0.01	0.00	0.00	0.01	0.02	0.52	0.06	0.27
Unknown			8.13			GL-RP-LC-QTOF-MS	ESI -		0.31	0.44	0.48	0.50	0.49	0.51	0.45	0.49	0.54	0.48	0.55	0.48
Unknown			9.09			GL-RP-LC-QTOF-MS	ESI -		0.13	0.58	0.85	0.05	0.07	0.06	0.06	0.00	0.00	0.00	0.00	0.00
Unknown			9.42			GL-RP-LC-QTOF-MS	ESI -		0.47	0.64	0.40	0.20	0.21	0.21	0.18	0.21	0.28	0.15	0.11	0.09
Unknown			9.42			GL-RP-LC-QTOF-MS	ESI -		0.38	0.52	0.34	0.13	0.15	0.15	0.12	0.13	0.16	0.09	0.07	0.06
Unknown			10.84			GL-RP-LC-QTOF-MS	ESI -		0.48	0.24	0.51	0.26	0.34	0.23	0.16	0.14	0.14	0.09	0.30	0.12
Unknown			8.69			GL-RP-LC-QTOF-MS	ESI -		0.10	0.25	0.13	0.41	0.38	0.35	1.02	0.92	0.15	0.09	0.12	0.12
Unknown			8.14			GL-RP-LC-QTOF-MS	ESI -		0.07	0.19	0.10	0.29	0.26	0.20	0.77	0.73	0.10	0.05	0.06	0.06

GL: global lipidomics, LC: liquid chromatography, QTOF-MS: quadrupole time-of-flight mass spectrometer.
