# Supplementary material for: Predicting the microalgae lipid profile obtained by supercritical fluid extraction using a machine learning model
Source: Front Chem. 2024 Oct 25;12:1480887. doi: 10.3389/fchem.2024.1480887 (PMC11543471; doi:10.3389/fchem.2024.1480887)
Supplement: Supplementary file 5 [file DataSheet2.docx]

**Supplementary Data 2**

**Table SD2.** Initial parameters for Grid-Search 5-fold Cross-Validation for each regression model.

| **Model** | **Initial Grid-Search Parameters** | **Final Parameters** |
| --- | --- | --- |
| **Lasso** | 'alpha': [0.0001, 0.001, 0.01, 0.1, 1] | 'alpha':0.0001 |
| **Gaussian Regression (GPR)** | 'kernel': RBF(length_scale=1.0),  'alpha': [1e-10, 1e-2, 1, 10],  'normalize_y': [True, False],  'n_restarts_optimizer': [0, 5, 10] | kernel': RBF(length_scale=66),  alpha': 0.063, '  'normalize_y': True,  'n_restarts_optimizer': 9, |
| **XG Boost (XGB)** | 'learning_rate': [0.1, 0.15, 0.2],  'n_estimators': [35, 50, 75, 100],  'max_depth': [5, 10, 15],  'subsample': [0.8, 0.9, 1.0],  'colsample_bytree': [0.8, 0.9, 1.0],  'min_child_weight': [1, 2, 3],  'gamma': [0, 0.1, 0.2],  'reg_alpha': [0, 0.01, 0.1],  'reg_lambda': [0.5, 0.75, 1, 1.5, 2.0] | 'learning_rate': 0.15,  'n_estimators': 35,  'max_depth': 14,  'subsample': 0.82,  colsample_bytree': 1.0,  'min_child_weight': 2,  'gamma': 0,  reg_alpha': 0.1,  'reg_lambda': 0.75 |
| **Random Forest Regressor (RFR)** | 'n_estimators': [500, 75, 100],  'max_depth': [None, 10, 15, 20],  'min_samples_split': [2, 5, 10],  'min_samples_leaf': [1, 2, 4],  'max_features': ['auto', 'sqrt', 'log2'],  'bootstrap': [True, False] | 'n_estimators': 50,  max_depth': 15,  'min_samples_split': 2,  'min_samples_leaf': 1,  'max_features': 'auto',  bootstrap': True |
| **Support Vector Regressor (SVR)** | 'kernel': ['rbf'],  'C': [0.1, 1, 10, 100, 1000, 10000],  'gamma': ['scale', 'auto', 1, 0.1, 0.01],  'epsilon': [0.01, 0.1, 0.2, 0.5, 1] | 'kernel': ['rbf'],  'C': 47500,  'gamma': 0.0095  'epsilon': 0.09 |
| **Artificial Neural Network (ANN)** | 'Input ': 32  'Dense 1': [21, 19, 17, 15, 13, 11]  'Dense 2': [13, 11, 9, 7, 5, 3]  'Output ': 1 | 'Input ': 32  'Dense 1': 13  'Dense 2': 7  'Output ': 1 |
