# Supplementary material for: Predicting the microalgae lipid profile obtained by supercritical fluid extraction using a machine learning model
Source: Front Chem. 2024 Oct 25;12:1480887. doi: 10.3389/fchem.2024.1480887 (PMC11543471; doi:10.3389/fchem.2024.1480887)
Supplement: Supplementary file 6 [file DataSheet1.docx]

**Supplementary Data 1**

**Table SD1.** RDKit Molecular descriptors selected for the machine learning regression models after cleaning and preprocessing data process

| **Descritor** | **Explanation** |
| --- | --- |
| qed | Quantitative Estimate of Drug-likeness |
| SPS | Surface Points Sum |
| MinPartialCharge | The minimum partial charge on any atom in the molecule |
| BalabanJ | topological index used to describe the branching. |
| HallKierAlpha | The Hall-Kier Alpha is a connectivity index. It utilizes the count of neighboring atoms bonded to an atom in the hydrogensuppressed graph |
| Ipc | Ipc index is the information for polynomial coefficients based information theory |
| PEOE_VSA7 | Partial Equalization of Orbital Electronegativities (PEOE) and Van der Waals Surface Area (VSA). 7 [0.10, 0.15) |
| PEOE_VSA8 | Partial Equalization of Orbital Electronegativities (PEOE) and Van der Waals Surface Area (VSA). 8 [0.15, 0.20) |
| PEOE_VSA9 | Partial Equalization of Orbital Electronegativities (PEOE) and Van der Waals Surface Area (VSA). 9: [0.20, 0.25) |
| PEOE_VSA11 | Partial Equalization of Orbital Electronegativities (PEOE) and Van der Waals Surface Area (VSA). 11: [-0.40, -0.30) |
| SMR_VSA3 | Molar Refractivity (SMR) and Van der Waals Surface Area (VSA). 3: [2.0, 2.5) |
| SMR_VSA9 | Molar Refractivity (SMR) and Van der Waals Surface Area (VSA). 9: [4.0, 4.5) |
| SlogP_VSA1 | LogP (octanol/water partition coefficient) and VSA. 1: [-0.40, -0.20) |
| MaxAbsEStateIndex | Maximum absolute value of the E-State (electrotopological state) |
| MinAbsEStateIndex | Maximum absolute value of the E-State (electrotopological state) |
| MinEStateIndex | Minimum E-State index for the atoms in the molecule |
| EState_VSA2 | based on the E-State index (electrotopological state) and VSA (Van der Waals Surface Area). 2: [0.10, 0.20) |
| EState_VSA3 | based on the E-State index (electrotopological state) and VSA (Van der Waals Surface Area). 3: [0.20, 0.30) |
| EState_VSA4 | based on the E-State index (electrotopological state) and VSA (Van der Waals Surface Area).  4: [0.30, 0.40) |
| EState_VSA6 | based on the E-State index (electrotopological state) and VSA (Van der Waals Surface Area). 6: [0.50, 0.60) |
| EState_VSA7 | based on the E-State index (electrotopological state) and VSA (Van der Waals Surface Area). 7: [0.60, 0.70) |
| VSA_EState4 | VSA (Van der Waals Surface Area) and E-State (electrotopological state) values in a specific way. 8: [-0.10, 0.00) |
| VSA_EState6 | VSA (Van der Waals Surface Area) and E-State (electrotopological state) values in a specific way. 6: [-0.30, -0.20) |
| VSA_EState8 | VSA (Van der Waals Surface Area) and E-State (electrotopological state) values in a specific way. 8: [-0.10, 0.00) |
| NumAliphaticHeterocycles | Number of aliphatic heterocycles |
| NumAromaticHeterocycles | Number of aromatic heterocycles |
| NumAromaticRings | Number of aromatic rings |
| fr_Al_COO | Number of aliphatic carboxyl groups |
| fr_ketone | Number of ketone groups |
